# Ultrasound‐targeted microbubble destruction mediates PDE5i/NO integration for cavernosum remodeling and penile rehabilitation

**DOI:** 10.1002/btm2.10568

**Published:** 2023-07-02

**Authors:** Wende Yang, Chen Qiu, Jiancheng Zhai, Wei Zhang, Chengwu Huang, Jun Shao, Jingke Zhang, Shigao Chen, Xiaoyan Miao, Peng Chen, Bo Wei, Jie Ren, Hongbo Wei

**Affiliations:** ^1^ Department of Gastrointestinal Surgery The Third Affiliated Hospital of Sun Yat‐sen University Guangzhou China; ^2^ Department of Medical Ultrasound, Laboratory of Novel Optoacoustic (Ultrasonic) Imaging The Third Affiliated Hospital of Sun Yat‐sen University Guangzhou China; ^3^ Department of Ultrasound The Second Affiliated Hospital Zhejiang University School of Medicine Hangzhou China; ^4^ Department of Radiology Mayo Clinic College of Medicine and Science Rochester Minnesota USA

**Keywords:** erectile dysfunction, microbubble, penile rehabilitation, UTMD

## Abstract

Erectile dysfunction (ED) caused by cavernous nerve injury (CNI) is refractory to heal mainly ascribed to the adverse remodeling of the penis induced by ineffectual microvascular perfusion, fibrosis, and neurotrophins scarcity in cavernosum. Phosphodiesterase type V inhibitors (PDE5i) have been regarded as an alternative candidate drug for avoiding penile neuropathy. However, the therapeutic efficacy is severely limited due to poor accumulation under systemic medication and endogenous nitric oxide (NO) deficiency in cavernosum. Herein, an innovative liposomal microbubble (MB) loaded with both Sildenafil (one of PDE5i) and NO was designed. Ultrasound‐targeted MB destruction (UTMD)‐mediated efficient release and integration erectogenic agents into corpus cavernosum with high biosafety. On a bilateral CNI rat model, the multifunctional MB‐cooperated UTMD improved microvascular perfusion in penis, simultaneously, alleviated hypoxia and oxidative stress, indicating successful activation of NO–cyclic guanosine monophosphate pathway. Also, evaluation of the endothelial/muscular composition, intracavernosal pressure, and neural integrity in the penis proved that coordinated intervention reversed the abnormal structural remodeling and promoted the recovery of functional erection. Our work demonstrates that MB loading Sildenafil and NO combined with UTMD hold great promise to “awaken” the efficacy of PDE5i in neurogenic ED, which provided a superior option for ensuring penile rehabilitation.

## 1 INTRODUCTION

Erectile dysfunction (ED), which is defined as an inability to achieve or maintain an erection for satisfactory sexual intercourse,^1^ is caused by a variety of pathological conditions, including vascular risk factors, neurological abnormalities, and hormonal disorders.^2^ Neurologic disorder is one of the most common causes of ED, and about 50%–90% patients suffering pelvic floor surgery may have ED due to the difficulty in avoiding damage to the cavernous nerve (CN),^3^ a branch of the pelvic autonomic nerve. It is reported that only a few of the neurogenic ED patients could obtain an erectile response after months or even years of recovery, moreover, it is extremely hard to achieve a functional recovery of the penis characterized by a completion of sexual intercourse and ejaculatory function for those “recovered” patients.^4^

Slow axonal growth (presynaptic mechanism) had been considered as a main reason for poor functional recovery in neurogenic ED, however, it has been observed that penis rehabilitation is not possible in neurogenic ED even under ideal regeneration of CN,^5^, ^6^ suggesting that simply focusing on presynaptic mechanism alone may not be sufficient to explain the difficulty in treatment of neurogenic ED. In recent years, penile responsiveness alterations (postsynaptic mechanisms) including vascular dysfunction, fibrosis, and neurotropism deficiency in the corpus cavernosum have been received an increasing attention in neurogenic ED.^5^, ^7^ The circulation of the microvascular system in the cavernous is impaired due to the chronic penile weakness and lack of nocturnal erection in neurogenic ED, resulting in continuous ischemia or hypoxia in the penis.^8^ The local pathological microenvironment further leads to diffuse corpus cavernosum fibrosis (CCF), which may appear as early as 1 week after CN injury.^6^ Once CCF progressively worsens and develops into diastolic and contractile dysfunction of the cavernous tissue, complete repair of the damaged CN, local injection therapy or even prosthetic implantation to restore erectile function will no longer be possible.^9^ In addition, a variety of neurotrophic factors (NFs), including glial cell‐derived neurotrophic factor which can be produced by the penis itself for CN protection and regeneration,^10^ but it may be completely interrupted in neurogenic ED due to structural and functional disruption of the penis. Therefore, delaying or even reversing the target organ lesions of neurogenic ED not only supports regeneration of CN, but also benefits for reconstruction of the penile tissue, which is a critical and non‐negligible part for penile function rehabilitation. However, a therapeutic strategy for target organ protection in neurogenic ED has not been completely explored.

Phosphodiesterase type V inhibitors (PDE5i) has been regarded as the first‐line treatment for ED in clinic, it can activate the nitric oxide (NO)–cyclic guanosine monophosphate (cGMP) pathway by inhibiting the breakdown of cGMP to induce an erectile response, that is, smooth muscle diastole of the penile corpus cavernosum,^11^ and thus promote oxygen supply to the corpus cavernosum by increasing oxygen‐rich arterial and avoid denervated reactive changes in the penis. Notably, the efficacy of PDE5i relies on the formation of endogenous NO, as a matter of fact, multiple factors contributing to neurogenic ED could result in reduced responsiveness in oral PDE5i therapy, including cavernous endothelial dysfunction and cavernous neuropathy,^12^, ^13^ which leads to insufficient bioavailable NO to raise sufficient cGMP above the threshold required for the penile erectile response. At the same time, the traditional oral administration of PDE5i have potential risks of systemic organ toxicities and complications (i.e., cardiovascular complications, even hearing loss and nonarteritic pre‐ischemic optic neuropathy) due to systemic drug distribution and repeated dosing, more importantly, it is less likely to be enriched in the penis even at higher conventional oral doses.^14^, ^15^ Therefore, introducing exogenous NO to “awaken” the efficacy of PDE5i in neurogenic ED while guaranteed lower dose but better enrichment of PDE5i in the penile corpus cavernosum will likely be a promising strategy for targeted organ protection and functional rehabilitation in neurogenic ED.

Ultrasonic microbubbles (MBs) are a kind of core‐shell structure consisting of a shell membrane and a gas core with micron or nano scale. Recent studies have shown that ultrasound‐targeted microbubble destruction (UTMD) technique is a safe and effective strategy for targeted drug delivery.^16^, ^17^ When MBs expose to ultrasound, they oscillate and collapse to produce cavitation effect in a short period of time which would increase the permeability of cells and blood vessel walls while releasing drug or gene delivery at a specific site.^18^ As a result, the drug or gas encapsulated in the MBs could be delivered into deep tissue and targeted cells, leading to an increase in local drug or gas concentration. Collectively, MBs combining with UTMD could be an ideal method for precise and effective drug (PED5i) and gas (NO) delivery to the penile corpus cavernosum.

In this study, we attempted to design an ultrasonic MBs co‐loaded with both NO and sildenafil which is the classical representative of PDE5i and the most commonly drug used for ED. Then, we aimed to verify the therapeutic efficacy of the MBs combining with UTMD in neurogenic ED through effectively delivering NO to penile corpus cavernosum and “awaken” the therapeutic efficacy of sildenafil in neurogenic ED.

## 2 MATERIALS AND METHODS

### 2.1 Preparation and characterization of MBs

All phospholipids including 18 mg 1,2‐dihexadecanoyl‐rac‐glycero‐3‐phosphocholine (DPPC), 3.5 mg distearoyl phosphatidyl ethanolamine polyethylene glycol 2000 (DSPE‐PEG2000), 1 mg diphenyl phosphoryl azide (DPPA) and 2 mg Sildenafil (HY‐15025, MCE) were dissolved in 4 mL chloroform in a round‐bottom flask. Afterwards, the solution was evaporated at 60°C for about 0.5 h to form a phospholipid structure. Maintaining at 60°C for 0.5 h, the phospholipid film was then hydrated using 4 mL phosphate‐buffered saline (PBS) to form Sildenafil‐loaded liposomes. Then, 0.5 mL Sildenafil‐loaded liposomes suspensions were put in a 1.5‐mL centrifuge tube and purged with different proportions of NO (0%, 20%, 40%, 60%, 80%, and 100%) and perfluoropropane (C3F8) gas mixture continuously to replace the air inside. The admixture was then mechanically vibrated for 45 s at 70 Hz using a dental amalgamator (YG‐10, ZOGEAR, China) to obtain final different types of Sil‐NO‐MBs. The diameters of the MBs were detected using the dynamic light scattering (DLS) method (90 Plus/BI‐MAS, Brookhaven Instruments, USA).

### 2.2 Extraction of corpus cavernosum smooth muscle cells

Corpus cavernosum smooth muscle cells (CCSMCs) were extracted by trypsin digestion and tissue attached culture method, respectively, as described before.^19^ Briefly, pentobarbital sodium (30 mg/kg) was used to anesthetize the male Sprague Dawley rats, subsequently sacrificed and the skin and fascia on the penis surface were quickly removed. The penile corpus cavernous tissue was fully exposed and sterilized with 75% alcohol, then washed with PBS and cut into small tissue pieces of 1–2 mm^3^. The small pieces were placed evenly in a 25‐cm^2^ culture flask (Corning, Corning, NY, USA) in 2 mL high‐glucose Dulbecco's modified Eagle medium, which contained 10% fetal bovine serum. To prevent floating of the tissue blocks, contact between the culture medium and the tissue blocks was avoided by keeping the culture flasks in an upright position for about 1 h, then gently turned over and cultured in the incubator when the tissue was fully fixed to the bottom. The tissue was discarded after the cells migrated out and onto the place, and cells were cultured for another 48–72 h. The second to fourth passage of cells were used for subsequent experiments. Immunofluorescence was used to identify the surface marker of CCSMCs.

### 2.3 Stability and contrast‐enhanced ultrasound ability of MBs in vitro

An agar mold with 12 holes of 1.5 cm in diameter and 5 cm in depth was used to evaluate the imaging ability of MBs in vitro. Briefly, 20 μL different NO/C3F8 proportions of Sil‐NO‐MBs were dissolved in PBS forming a final volume of 1 mL suspensions. The solutions were then added into the holes and contrast‐enhanced ultrasound (CEUS) were performed at 0, 30, 60, 90, and 120 min. Images were obtained by using a clinical ultrasound (US) imaging system (Logiq E9, GE Healthcare, USA) equipped with a ML6‐15 probe with the following CEUS imaging parameters: a frequency of 11 MHz, a mechanical index of 0.11, and an CEUS gain of 24 db. After all the images were collected, we analyzed the signal intensity quantitatively with Image J software off‐line. The region of interest (ROI) is darker, that is, the intensity signal is weaker, which means the microbubble has suffered destruction; A brighter ROI indicates a stronger intensity signal, indicating that the integrity of the microbubble has been preserved.

### 2.4 Analysis of cellular activity under UTMD intervention

CCSMCs in logarithmic growth phase were inoculated in 96‐well plates (5 × 10^3^ cells/well). Sil‐NO‐MBs were added to the CCSMCs culture medium at dilution ratios of 1:10, 1:100, 1:1000, and 1:2000, respectively, and UTMD was performed with the assistance of ultrasonic guide pads as the experimental group. The parameters of UTMD were: ultrasound frequency = 1 MHz; duty cycle = 50%; ultrasound intensity = 2 w/cm^2^; ultrasound irradiation time was 2 min. The survival of cells was detected using CCK8 kit (Beyotime, C0038) after 4 and 8 h of intervention, respectively, and the absorbance at 450 nm was read using a BioTek multifunctional enzyme marker.

### 2.5 In vivo fluorescence imaging

The near‐infrared fluorescent dyes 1,1′‐dioctadecyl‐3,3,3′,3′‐tetramethylline‐ethyl trithiocyanate (DiR) were used instead of Sildenafil to prepare DiR‐NO‐MBs. UTMD parameters: ultrasound frequency = 1 MHz; duty cycle = 50%; ultrasound intensity = 2 w/cm^2^; ultrasound irradiation time was 2 min. The images were captured and quantified using a small animal imaging system (In vivo FX PRO, BRUKER).

### 2.6 Establishment of neurogenic ED model in rats and treatments

All animal experimental procedures were approved by the Hospital Animal Research Committee of the Third Affiliated Hospital of Sun Yat‐sen University (experimental animal ethics number: 00308751). Adult male Sprague Dawley rats (10 weeks old, 250–300 g) were purchased Sja Biotechnology Co., Ltd (Guangdong, China). All rats were housed in specific pathogen‐free conditions, with a 12/12‐h light/dark cycle, a temperature of 24 ± 2°C, humidity between 30% and 70%, and free access to food and water.

The neurogenic ED model was constructed as described previously. Briefly, rats were anesthetized with pentobarbital sodium (30 mg/kg) and the prostate was exposed through a ventral midline incision, with the posterior lateral CN and the pelvic mass ganglion (MPG) can be easily identified. Rats were subjected to sham or neurogenic ED modeling surgery and randomly divided equally into five groups: sham group, exposure and isolation of CN without clamping (Sham); the remaining four groups were isolated using vascular forceps followed by clamping with serrated forceps for 2 min and received the following interventions on days 3, 6, 9, and 12 after surgery: tail vein injection of 0.1 mL phosphate buffer, PBS group (BCNI + PBS), tail vein injection of Sildenafil, Sil group (BCNI + Sil), tail vein injection of Sil‐NO‐MBs, MBs group (BCNI + Sil‐NO‐MBs), and tail vein injection of Sil‐NO‐MBs combined with UTMD at the penile site, UTMD group (BCNI + Sil‐NO‐MBs + UTMD).

### 2.7 Measurement of intracavernosal pressure and mean arterial pressure

The erectile function was measured at the end of the intervention treatment in each group, that is, the 14th day before the termination of the animal. As previously reported,^20^ in brief, after anesthetizing the rats with sodium pentobarbital, the bilateral CN was exposed through the original ventral midline incision; the right carotid artery was fully exposed through an approximately 3‐cm‐long midline incision in the neck. A 25‐caliber butterfly needle filled with heparinized saline (200 IU/mL) connected to a pressure transducer was inserted into the left penile corpus cavernosum to record intracavernosal pressure (ICP). The PE‐50 tube connected to the tip of 25‐gauge butterfly needle was inserted into the right carotid artery to record mean arterial pressure (MAP). Both value of ICP and MAP were recorded from data acquisition system (BL420N, Chengdu Taimeng Technology Co., Ltd, China). CN was stimulated directly by bipolar stimulation electrodes (probe diameter 2 mm, spacing 1 mm). Stimulation parameters were: 1.5 mA, 20 Hz, pulse width 0.2 ms, duration 50 s. Penile erectile function was assessed by recording maximum ICP (ICPmax) and total ICP/total MAP.

### 2.8 Super‐resolution ultrasound localization microscopy data acquisition and analysis

The penile microcirculation was measured at the end of the intervention treatment in each group, that is, the 14th day before the termination of the animal. Rats were food‐deprived for 8–12 h in advance to avoid alterations in splanchnic hemodynamics and were anesthetized with an intraperitoneal injection of pentobarbital sodium (30 mg/kg), and then placed on a warming pad at 37°C to maintain body temperature. US imaging was performed using a VisualSonics Vevo 3100 high‐frequency imaging system (FUJIFILM VisualSonics Inc., Toronto, ON, Canada) and a linear array probe (MX500D, FUJIFILM VisualSonics Inc.) transmitting at 40 MHz. B‐mode and Doppler images were first acquired for reference. And then line‐by‐line focused B‐mode imaging was used for ultrasound localization microscopy (ULM) data acquisition in an RF data mode with a frame rate of 592 Hz at 10% transmitted acoustic power. About 10 in‐phase quadrature (IQ) data sets, each containing 1000 frames of ultrasound images, were collected right after a 0.2 mL bolus injection of MB solution (Bracco, Milan, Italy) via intravenous injection. The captured IQ data were processed offline to reconstruct ULM images with a pixel resolution of 6 μm in both spatial dimensions using customized ULM processing techniques detailed in previously.^21^

### 2.9 Hematoxylin and eosin, Masson's trichrome, and immunohistochemistry staining

Freshly dissected penises (mid‐axis portion) from each group of rats were fixed in 4% paraformaldehyde solution for 24 h and then dehydrated in a graded ethanol series. The dehydrated samples were embedded in paraffin and sectioned at 5 μm thickness parallel to the short axis of the penis, followed by hematoxylin and eosin staining or Masson's trichrome staining. In immunohistochemical (IH) analysis, the sections were dewaxed with xylene, hydrated with an ethanol series, and then placed in antigen repair solution and subjected to antigen repair at high temperature in a microwave oven. Subsequently, all sections were closed with 5% bovine serum albumin (BSA) for 60 min and incubated with primary antibodies at 4°C overnight. Next, each section was incubated with horseradish peroxidase (HRP)‐labeled secondary antibody at room temperature for 1 h. The sections were incubated using a DAB kit (Servicebio, Wuhan, China) to form colors, stained with hematoxylin, and images were captured using a fully automated digital section scanning system (PANNORAMIC MIDI II, China). The primary antibodies used for IH staining are listed in Table S3.

### 2.10 Immunofluorescence staining and histological analysis

The obtained penis and MPG paraffin sections were dewaxed in xylene, sequentially immersed in various levels of ethanol for hydration, then placed in antigen repair solution and subjected to high temperature in a microwave oven. All sections were closed with 5% BSA for 60 min and incubated with the corresponding primary antibody at 4°C overnight. After overnight incubation, secondary antibodies (1:500; Affinity) were applied and incubated for 60 min at room temperature, followed by 4',6‐diamidino‐2‐phenylindole (DAPI) (0.5 μg/mL; Invitrogen, Carlsbad, CA) staining. Images were obtained using a LSM880 laser confocal microscope (Zeiss, Germany) and further processed using ImageJ software. The primary antibodies used for immunofluorescence staining are listed in Table S3.

### 2.11 Western blot analysis

Penile tissue samples were lysed with radio immunoprecipitation assay (RIPA) buffer (50 mM Tris–HCl [pH 7.4], 150 mM NaCl, 0.1% sodium dodecyl sulfate [SDS], 1% Triton X‐100, 1% sodium deoxycholate, and 2 mM EDTA [pH 8.0]) containing protease inhibitor (Beyotime, P1005) and phosphatase inhibitor (Beyotime, P1081). The bicinchoninic acid assay (BCA) kit (P0010S, Beyotime) was used to determine the protein concentration. Tissue lysates containing 30 mg of protein were electrophoresed by SDS polyacrylamide gel electrophoresis and then transferred to polyvinylidene fluoride membranes (Millipore, Billerica, MA). Membranes were blocked with 5% (v/w) skim milk (diluted with TBS containing 0.1% Tween‐20) and incubated sequentially with primary and secondary antibodies. GAPDH or β‐actin was used as the loading control. Protein bands were visualized using Tanon 5200 (Tanon Technology Co., Ltd., Shanghai, China). Protein bands were quantified using Image J software. Unclipped blots are provided in the source data file. Specific information on the primary antibody used for Western Blot is provided in Table S3.

### 2.12 RNA extraction and quantitative real‐time PCR

Total RNA was extracted from fresh penile tissue using TRIzol (Ambion) according to the manufacturer's instructions, and the concentration and purity of total RNA was determined using a nanodrop spectrophotometer. Reverse transcription was performed using the kit PrimeScript™ RT Master Mix (Perfect Real Time; RR036A, Takara). RNA extraction and quantitative real‐time PCR (RT‐qPCR) was then performed using SYBR Green Premix Pro Taq HS qPCR Kit (AG11701, Accurate Biotechnology [Hunan] Co., Ltd) on an ABI7500 instrument (Thermo Fisher, USA). GAPDH was used as an internal control. Primers used for RT‐qPCR are shown in Table S1.

### 2.13 Tunel assay

Mid‐penile tissue was fixed and embedded with optimal cutting temperature compound (OCT) compound (Servicebio, G6059), cut to 5 μm thickness. The tissue was then permeabilized with PBS containing 0.25% Triton TMX‐100 and stained with the in‐situ cell death assay kit (Servicebio, G1502‐50T) according to the manufacturer's instructions. The TUNEL+ cells/Hoechst 33342+ cells ratio was quantified using ImageJ software.

### 2.14 Enzyme‐linked immunosorbent assay

Briefly, 200 mg of fresh tissue was collected at the end of the intervention in each group of rats to make supernatant. The concentration of cGMP was measured using a rat Enzyme‐linked immunosorbent assay (ELISA) kit (Wuhan mmbio Co., LTD., Wuhan, China) according to the manufacturer's instructions. The absorbance at 450 nm was read using a BioTek multifunctional enzyme marker.

### 2.15 Statistical analysis

The data which correspond to normal distribution are expressed as mean ± standard deviation (SD), otherwise expressed as Median with interquartile range. In the comparison between the two groups, the unpaired two‐tailed Student's *t*‐test or following Welch's correction were used to analyze the data that fit the normal distribution, while the Mann–Whitney test was used to analyze the data that did not fit. Statistical analysis was performed using GraphPad Prism 7 software (GraphPad Software, San Diego, CA). All data were repeated at least three times. Values of *p* < 0.05 were considered statistically significant.

## 3 RESULTS

### 3.1 Preparation and characterization of Sil‐NO‐MBs

**FIGURE 1:**
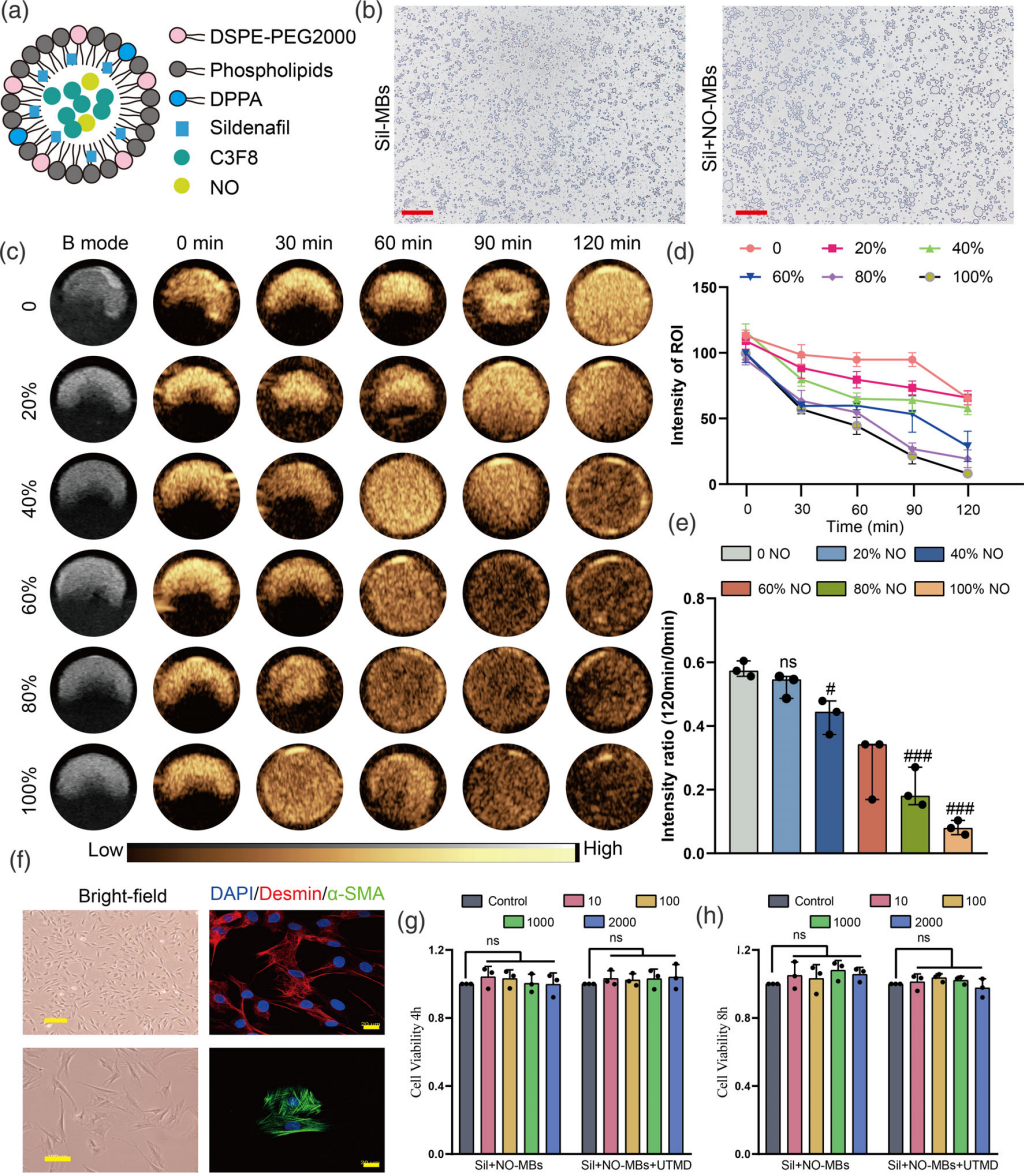
Preparation and characterization of MBs. (a) The design scheme of Sil‐NO‐MBs. (b) Observation of Sil‐MBs and Sil‐NO‐MBs using light microscopy, Scale bar = 50 μm. (c) Representative in vitro ultrasound images of Sil‐NO‐MBs. (d) Quantitative analysis of intensity signal of Sil‐NO‐MBs with different NO proportions at different time points (*n* = 3 per group). (e) The clearance ratio of Sil‐NO‐MBs in each group (*n* = 3 per group). (f) Observation of CCSMCs using light microscopy (top, scale bar = 200 μm; bottom, scale bar = 100 μm) and confocal scanning microscopy (scale bar = 20 μm), CCSMCs were stained with DAPI (blue fluorescence), Desmin (red fluorescence), α‐SMA (green fluorescence), and the images were merged. (g,h) Analysis of cell activity of CCSMCs cultured with different concentration of Sil NO MBs in 4 and 8 h later by UTMD or non‐UTMD (*n* = 3 per group). #*p <* 0.05; ##*p <* 0.01; ###*p <* 0.001. CCSMCs, corpus cavernosum smooth muscle cells; DAPI, 4',6‐diamidino‐2‐phenylindole; MBs, microbubbles; NO, nitric oxide; ns, nonsignificance; ROI, region of interest; Sil, sildenafil; UTMD, ultrasound‐targeted microbubble destruction; α‐SMA, α‐smooth muscle antibody.

The design scheme of Sil‐NO‐MBs is shown in Figure 1a, which has a circular spherical shape under light microscopy (Figure 1b). To determine the appropriate ratio of NO in MBs, we investigated the stability of MBs with different NO ratios of 0%, 20%, 40%, 60%, 80%, and 100% in vitro, respectively. The clearance time and signal intensity of MBs with increasing proportion of NO at different time points of 0, 30, 60, 90, and 120 min were compared to MBs without NO (Figure 1c,d). These results indicated that the stability of MBs decreased with the increase of NO proportion. In order to more intuitively reflect the stability of microbubbles with different NO ratios, we analyzed the signal intensity ratio of the microbubbles with different NO ratios after 2 h of preparation and the initial state (i.e., 0 h), as shown in Figure 1e, the best stability was achieved at 20% NO ratio, and the microbubble integrity was close to 60%, which was not significantly different from the best stability‐all inert gas C3F8 (0% NO) microbubbles. Therefore, MBs with a NO proportion of 20% were used in this study considering their stability and CEUS ability were closest to those without NO. Similar to previous studies in our group, the drug encapsulation efficiency of MBs showed the drug loading content and drug loading efficacy was about 4% and 50%, respectively, when putting in 2 mg of Sil (Figure S1). Therefore, we prepared the drug and gas co‐loaded MBs with 2 mg of Sil and 20% of NO for the best feasibility. We measured the particle size of both microbubbles using the DLS method. The diameters of Sil‐MBs were 1.82 ± 0.12 μm, while the diameters of Sil‐NO‐MBs were 1.44 ± 0.19 μm (*p* < 0.05; Figure S2), suggesting that the additional addition of NO did not affect the basic properties of the Sil‐loaded MBs.

In order to assess the toxicity and safety of MBs combining with UTMD application in vitro, we extracted CCSMCs from rat penis, which charactered as shuttle‐shaped, and positive expression of Desmin and α‐SMA proteins (Figure 1f). Freshly prepared Sil‐NO‐MBs of continual concentration were added to CCSMCs with non‐UTMD as the control group and UTMD as the experimental group. As a result, no significant differences in cell activity were observed between the two groups at 4 and 8 h after treatment, suggesting that Sil‐NO‐MBs did not have obvious cytotoxicity, and the UTMD parameters used in this experiment did not affect the cell activity (Figure 1g,h).

### 3.2 Increased drug and gas release and integration into cavernosum from Sil‐NO‐MBs via UTMD

By using coumarin mimicking Sil, we prepared Coumarin‐NO‐MBs to evaluate drug releasing efficacy with application of UTMD in vitro. As a result, the UTMD group showed stronger green fluorescence than non‐UTMD group, suggesting that the potential increase in cell membrane permeability which further facilitated the internalization of the drug after UTMD (Figures S3a,c). Similarly, it was found by DAF‐FM DA (NO fluorescent probe) assay that CCSMCs showed higher intracellular NO concentrations after UTMD compared to the non‐UTMD group (Figure S3b,d). The above in vitro results suggest that MBs combined with UTMD could promote the releasing and cellular uptaking of drugs and gases.

**FIGURE 2:**
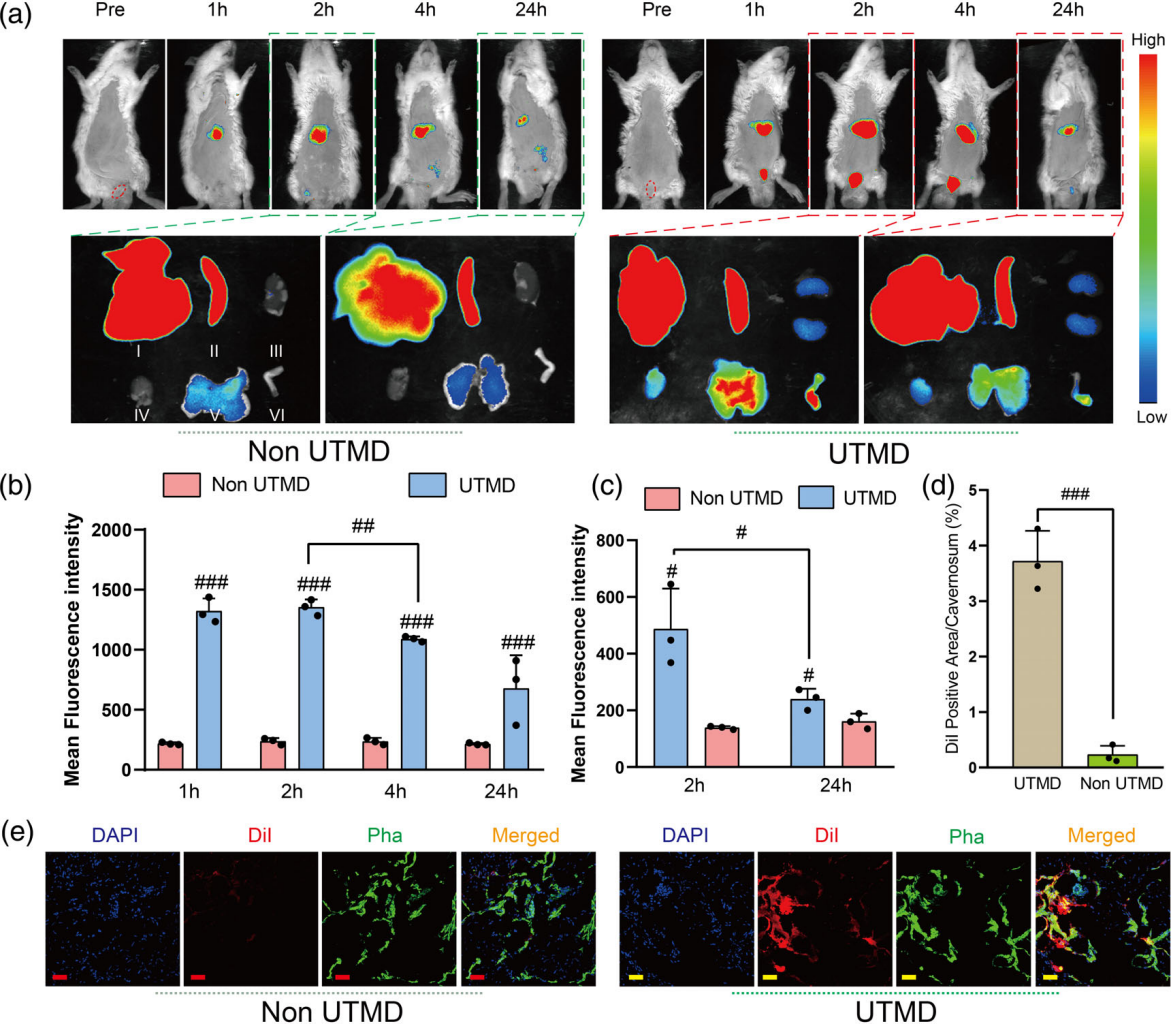
Drug and gas release and integration in the corpus cavernosum by UTMD. (a) In vivo fluorescence imaging at different time points after intravenous injection of Dir‐NO‐MBs with or without UTMD exposure at the penis. The penis is circled with a red dashed line. Images of organs excised at 2 and 24 h after injection was indicated by blue and red dashed lines, where I indicates liver, II indicates spleen, III indicates kidney, IV indicates heart, V indicates lung, and VI indicates penis. (b,c) Quantification of fluorescence intensity of penis in vivo and ex vivo respectively (*n* = 3 per group). (d) Quantification of fluorescence intensity of DiI in cavernosum (*n* = 3 per group). (e) DiI (red fluorescence) and pha staining (green fluorescence) for smooth muscle in the penile after intravenous injection of DiI‐NO‐MBs by UTMD or non‐UTMD intervention. Scale bar = 50 μm. #*p <* 0.05; ##*p <* 0.01; ###*p <* 0.001. Dir, 1,1′‐dioctadecyl‐3,3,3′,3′‐tetramethylline‐ethyl trithiocyanate; MBs, microbubbles; NO, nitric oxide; UTMD, ultrasound‐targeted microbubble destruction.

To simulate the distribution of drugs in animals, DIR‐NO‐MBs were prepared. Fluorescence intensity and distribution were observed in rats before and 1, 2, 4, and 24 h after the intervention. It was confirmed that the penile fluorescence intensity was significantly stronger in the DIR‐NO‐MBs + UTMD intervention group than in the non‐UTMD group (Figure 2). Although the penile fluorescence intensity in the DIR‐NO‐MBs + UTMD group was significantly lower after 24 h compared with 2 h, it still showed higher tendency than that in the non‐UTMD group (Figure 2a–c), indicating the sufficient drug retention in penis cavernosum for at least 24 h after UTMD.

To better depict the location of drug in the penis after UTMD, we fabricated NO‐MBs marked by DiI (red fluorescence) and labeled cavernous smooth muscle with pha (green fluorescence). Stronger intensity of red fluorescence in penile sections was observed in the DiI‐NO‐MBs + UTMD group and DiI was extensively colocalize with cavernous smooth muscle (Figure 2d,e), indicating that DiI was efficiently released from disrupted DiI‐NO‐MBs and diffused into the penile interstitium with the application of UTMD, thus exerting a potential targeting therapeutic effect.

### 3.3 Enhanced erectile function by Sil‐NO‐MBs combined with UTMD

**FIGURE 3:**
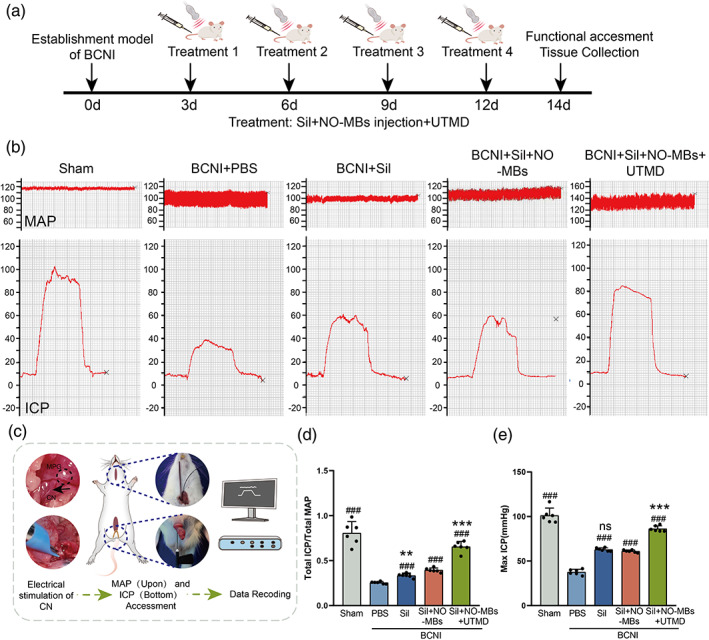
Enhanced erectile function by Sil‐NO‐MBs combined with UTMD. (a) Schematic diagram of a strategy based on Sil‐NO‐MBs combined with UTMD at the penis for the treatment of neurogenic ED. (b) Evaluation of erectile function. Top: Representative ICP on electrostimulation of the CN. Bottom: Representative pressure of the MAP of each group. (c) Abbreviated schematic diagram of the erectile function assessed by electrical stimulation of the CN to induce an erectile response in the penis. (d) Ratio of total ICP (AUC) to total MAP (AUC) (*n* = 6 per group). (e) Bar graphs represent the maximum ICP detected at 14 days of each group (*n* = 6 per group). PBS versus other groups: #*p <* 0.05, ##*p <* 0.01, ###*p <* 0.001. MBs versus Sil and UTMD group: **p < 0.01, ***p < 0.001. AUC, area under curve; CN, cavernous nerve; ED, erectile dysfunction; ICP, intracavernous pressure; MAP, mean arterial pressure; MBs, microbubbles; NO, nitric oxide; PBS, phosphate‐buffered saline; Sil, sildenafil; UTMD, ultrasound‐targeted microbubble destruction.

The therapeutic effect of Sil‐NO‐MBs combined with UTMD in a rat model of neurogenic ED was evaluated, and the intervention scheme is schematically shown in Figure 3a. The erection in each group was evaluated by measuring the ICP and MAP in different groups. As shown in Table S2, ICP_total_/MAP_total_ (0.81 ± 0.13 vs. 0.25 ± 0.01) and ICP_max_ (101.12 ± 8.38 vs. 37.67 ± 3.10) were significantly lower for PBS group compared to sham group, suggesting that the neurogenic ED model was successfully constructed. Generally, the penis in the UTMD group exhibited an ICP_total_/MAP_total_ ratio and ICP_max_ value similar to that in the sham group, and the penis in the other injection groups showed a much lower of both ICP_total_/MAP_total_ and ICP_max_ than the sham group (Table S2). Specifically, the ICP_total_/MAP_total_ ratio of the UTMT group (0.65 ± 0.06) were closer to that of the sham group (0.81 ± 0.13), and the ICP_total_/MAP_total_ values were higher than those of the other groups and much greater than those of PBS group (Figure 3b). The changes of ICP_max_ values in each group were similar to the ICP_total_/MAP_total_ ratio (Table S2 and Figure 3d,e). These results suggested a positive effect of UTMD application on erectile function.

### 3.4 Sil‐NO‐MBs combined with UTMD improve penile microcirculation

**FIGURE 4:**
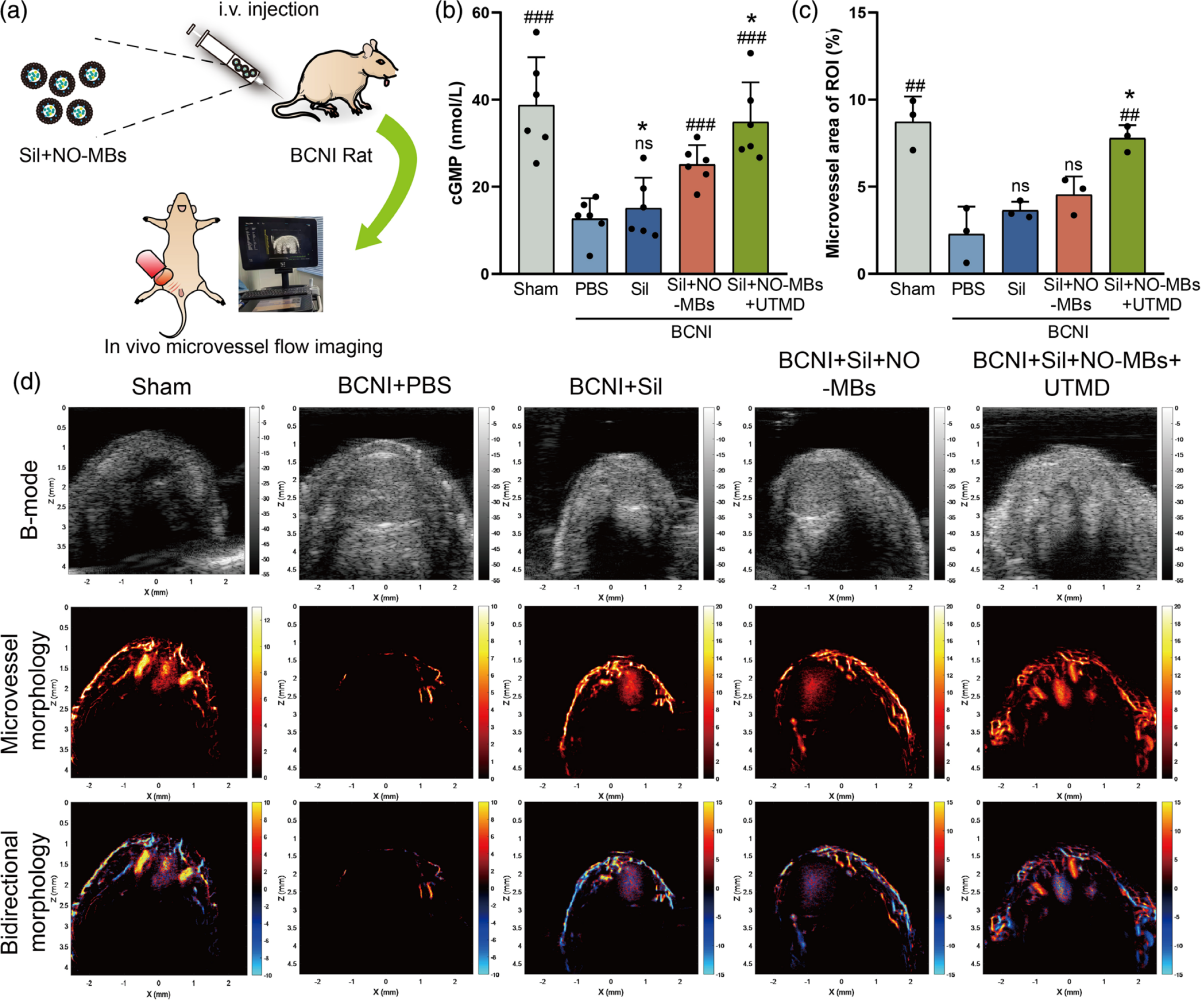
Sil‐NO‐MBs combined with UTMD improve penile microcirculation. (a) Schematic diagram of super‐resolution ultrasound microperfusion imaging of the penis. (b) Expression of cGMP in each group were analyzed at day 14 postoperation (*n* = 6 per group). (c) Quantitative analysis of microvascular area of cavernosum (*n* = 3 per group). (d) Representative images in each group of ULM. PBS versus other groups: #*p <* 0.05, ##*p <* 0.01, ###*p <* 0.001. MBs versus Sil and UTMD group: *p < 0.05. cGMP, cyclic guanosine monophosphate; MBs, microbubbles; NO, nitric oxide; PBS, phosphate‐buffered saline; Sil, sildenafil; ULM, ultrasound localization microscopy; UTMD, ultrasound‐targeted microbubble destruction.

Endothelial dysfunction is an important cause of poor penile structural remodeling in neurogenic ED,^22^ and timely restoration of penile microperfusion is critical for penis protection. The rats in each group were injected with Sil‐NO‐MBs intravenously at the endpoint of intervention to observe microcirculation detecting by ULM (Figure 4a). Penile microperfusion exhibited mild recovery in the neurogenic rats of Sil and MBs group compared to the PBS group (Figure 4c,d). As expected, UTMD group showed the best performance in regaining microperfusion compared to other groups (Figure 4c,d). In addition, as a downstream target molecule of PDE5i/NO, cGMP contributes mainly to penile microperfusion through mediating CCSMCs diastole.^23^ By ELISA analysis, it was found that the expression of cGMP was higher in both Sil and UTMD group compared to PBS group, but the UTMD group showed the best elevation (Figure 4b). These findings could be attributed to the targeted released of Sil and NO in penis simultaneously, and successful activation of NO/cGMP pathway, thus improved penile microperfusion over time.

**SCHEME 1:**
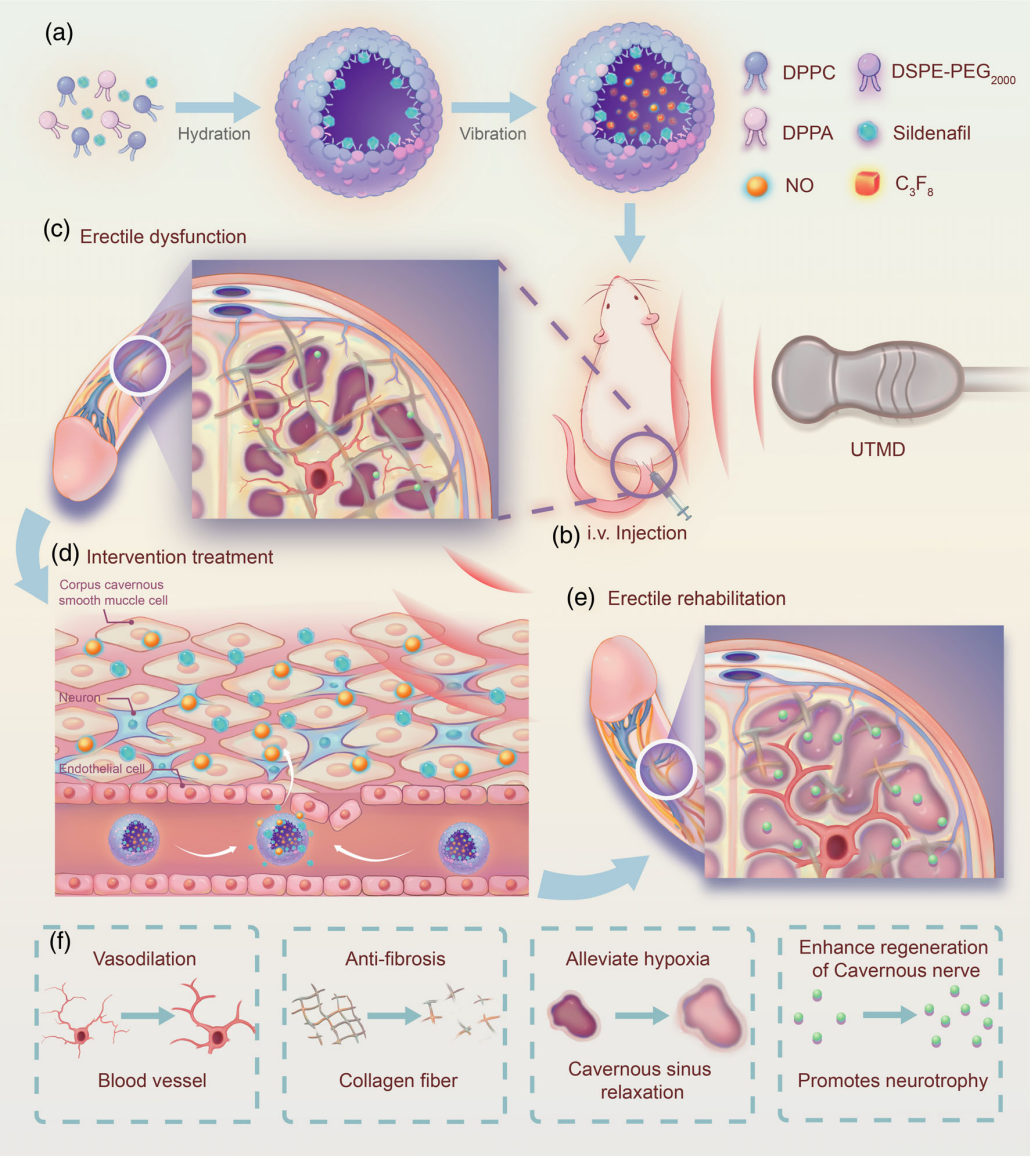
Schematic diagram of the therapeutic process of Sil‐NO‐MBs combined with UTMD. Schematic diagram of a proposed mechanism showing how Sil‐NO‐MBs combined with UTMD restored erectile function in neurogenic rat. MB, microbubble; NO, nitric oxide; Sil, Sildenafil; UTMD, ultrasound‐targeted microbubble destruction.

### 3.5 Sil‐NO‐MBs combined with UTMD protects endothelial integrity

**FIGURE 5:**
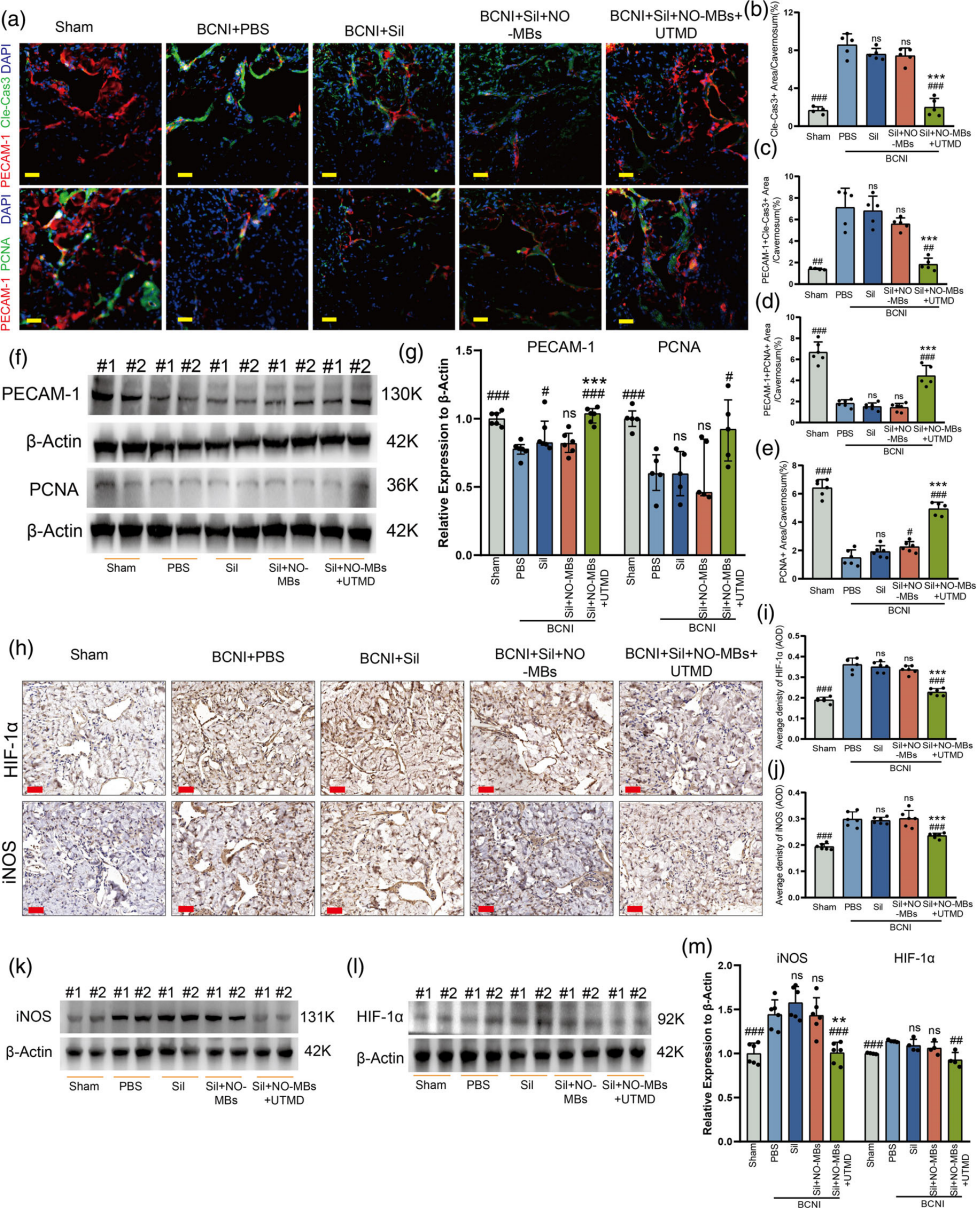
Sil‐NO‐MBs combined with UTMD protects endothelial integrity and alleviated cavernous hypoxia and oxidative stress. (a) PECAM‐1 (red) and Cle‐Cas3 (green) or PECAM‐1 (red) and PCNA immunostaining in cavernosum. Nuclei were labeled with DAPI (blue), scale bars: 50 μm. (b) Quantitative analysis of Cle‐Cas3‐positive area of cavernosum (*n* = 4–5 per group). (c) Quantitative analysis of both Cle‐Cas3‐ and PECAM‐1‐positive area of cavernosum (*n* = 4–5 per group). (d) Quantitative analysis of both PCNA‐ and PECAM‐1‐positive area of cavernosum (*n* = 5–6 per group). (e) Quantitative analysis of PCNA‐positive area of cavernosum (*n* = 5–6 per group). (f) Representative Western blots for PECAM‐1 and PCNA at 2 weeks after treatment. (g) Normalized band intensity values for both PECAM‐1 and PCNA (*n* = 5–6 per group). (h) Representative HIF‐1α and iNOS immunohistochemistry staining of corpus cavernosum obtained at 14 days postoperation in each group, Scale bars: 100 μm. Quantitative analysis parameter, AOD results of (i) HIF‐1α, (j) iNOS in each group (*n* = 6 per group). Representative Western blots for (k) iNOS, (l) HIF‐1α. (m) Normalized band intensity values (*n* = 6 per group). PBS verses other groups: #*p <* 0.05, ##*p <* 0.01, ###*p <* 0.001. MBs verses Sil and UTMD group: *p < 0.05, **p < 0.01, ***p < 0.001. AOD, average optical density; Cle‐Cas3, Cleaverd‐Caspase3; DAPI, 4',6‐diamidino‐2‐phenylindole; HIF‐1α, hypoxia inducible factor‐1α; iNOS, inducible nitric oxide synthase; MBs, microbubbles; NO, nitric oxide; PBS, phosphate‐buffered saline; PCNA, proliferating cell nuclear antigen; PECAM‐1, platelet/endothelial cell adhesion molecule 1; Sil, sildenafil; UTMD, ultrasound‐targeted microbubble destruction.

Double labeling of cavernous tissue with PECAM‐1 which is a specific endothelial cell marker and cleaved caspase‐3 which is an apoptosis marker showed that the numbers of apoptotic cells in cavernous endothelial cells (CECs) in the Sil and MBs group were close to that in the PBS group (Figure 5a–c). The apoptotic CECs were clearly scarcer in the UTMD group than in other injection groups (Figure 5a–c). To further verified whether the increase in CECs content correlated with cell proliferation, we assessed the number of CECs that stained positive for proliferating cell nuclear antigen (PCNA), a nuclear protein indicative of cell proliferation. The expression of PCNA were improved in the UTMD than in the PBS, Sil, MBs groups significantly (Figure 5a,d,e). In addition, the PCNA‐positive CECs were risen in the UTMD group which could be restored to the level of the sham group compared with that of the other groups (Figure 5a,d,e), which may, on the one hand, originate from the trophic effect of oxygen‐rich blood brought by cavernous smooth muscle diastole, in addition, Sil and NO alone have potential endothelial protective and vasodilatory effects.^24^, ^25^ Furthermore, Western blot assays also showed that the expression of PECAM‐1 and PCNA were higher expression in the UTMD and sham group compared with the other groups (Figures 5f,g, S5, and S6). These results indicated that the cavernosum in the UTMD group achieved improved microperfusion and endothelial protection.

### 3.6 Alleviation of hypoxia and oxidative stress in the penile corpus cavernosum by Sil‐NO‐MBs combined with UTMD

The expression of hypoxia inducible factor‐1α (HIF‐1α) was assessed by IH staining in hypoxic environment. The results indicated that the expression levels of HIF‐1α in the UTMD group decreased significantly in comparison to that of the PBS, Sil, and MBs group (Figure 5h,i). In addition, cavernous oxidative stress induced by penile hypoxia is an important factor in exacerbating neurogenic ED.^26^ Inducible nitric oxide synthase (iNOS), a key indicator of oxidative stress status, plays an important role in promoting the progression of organ inflammation and fibrosis.^27^ As shown in Figure 5h,j, the expression of iNOS in UTMD group was significantly reduced compared with other treatment groups, and the Western blot assay were accordant with the results of the IH assay (Figures 5k,l, S7, and S8), indicating that the UTMD group effectively improved cavernous oxidative stress and protected erection‐related tissue structures, which is also consistent with the study that inhibition of iNOS improved ED in type 1 diabetic rats.^28^

### 3.7 Sil‐NO‐MBs combined with UTMD promoted the secretion of neurotrophic factors in cavernous and protected CNs

**FIGURE 6:**
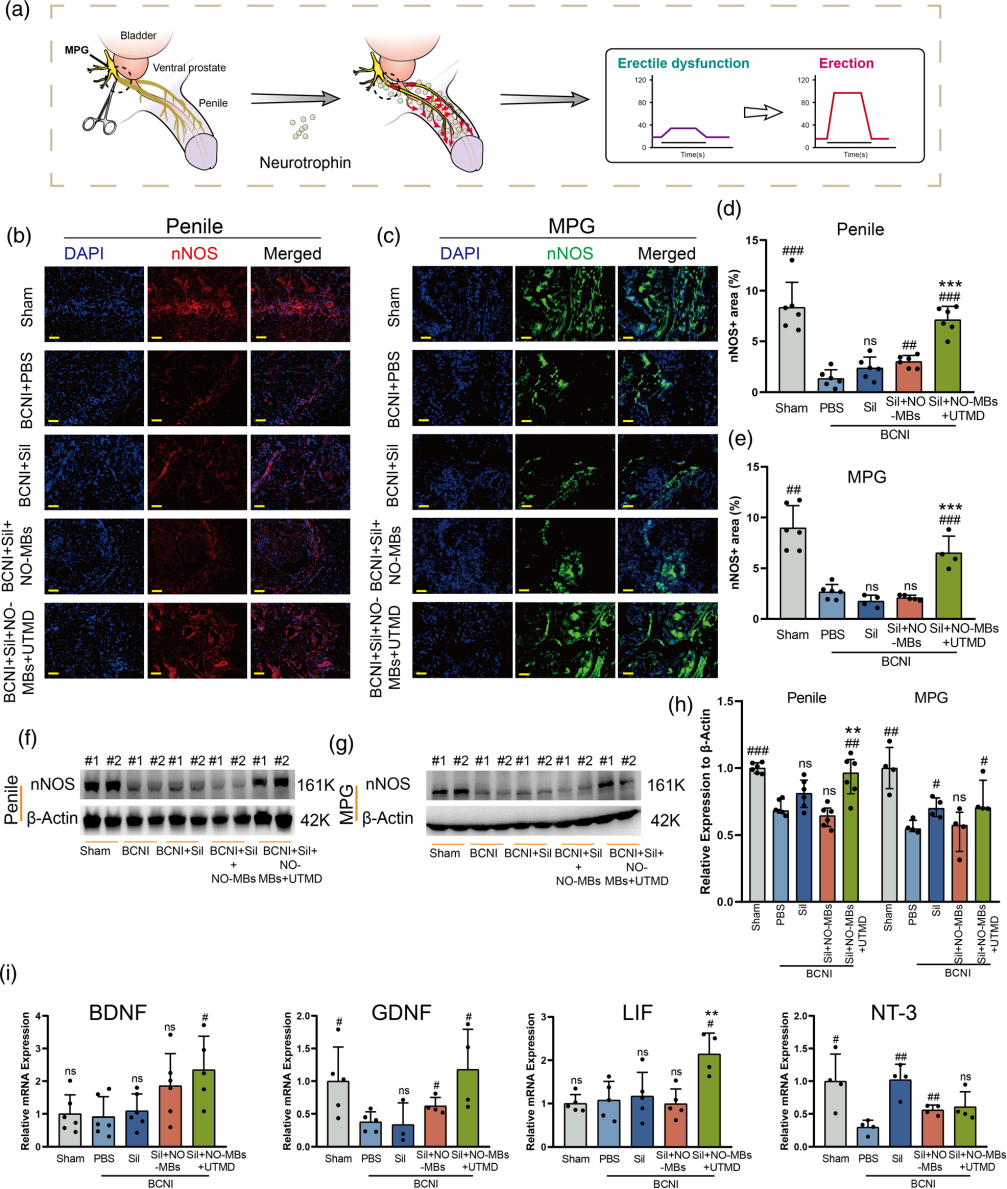
Sil‐NO‐MBs combined with UTMD promoted the secretion of neurotrophic factors in cavernous and protected cavernous nerves. (a) Schematic illustrating the protection of CN through retrogradely transport to MPG of neurotrophic factors producing by penis. (b,c) nNOS (red) in penis tissue and nNOS (green) in MPG from Sham or BCNI rat 2 weeks after a single intracavernous injection of PBS, Sil, Sil‐NO‐MBs, or Sil‐NO‐MBs + UTMD. Nuclei were labeled with DAPI (blue). Scale bars: corpus cavernosum, 100 μm; MPG, 100 μm. (d,e) Quantification of the nNOS in both cavernous tissue and MPG by ImageJ (*n* = 4–6 per group). Representative Western blots for nNOS in penile (f) and MPG (g). (h) Normalized band intensity values (*n* = 4–6 per group). (i) The mRNA's expression of BDNF, GDNF, LIF, NT‐3 in the cavernosum 2 weeks after intervention in each group (*n* = 4–6 per group). PBS versus other groups: #*p <* 0.05, ##*p <* 0.01, ###*p <* 0.001. MBs versus Sil and UTMD group: *p < 0.05, **p < 0.01, ***p < 0.001. BDNF, brain‐derived neurotrophic factor; CN, cavernous nerve; DAPI, 4',6‐diamidino‐2‐phenylindole; GDNF, glial cell‐derived nutrient factor; LIF, leukemia inhibitory factor; MPG, main pelvis ganglion; nNOS, neurotype nitric oxide synthase; NO, nitric oxide; NT‐3, neurotrophin‐3; Sil, sildenafil; UTMD, ultrasound‐targeted microbubble destruction.

Functional penis can produce NFs that nourish the CN by retrogradely transport to MPG that is beneficial for a functional erection,^10^ as shown schematically in Figure 6a. The number of intact neurotype nitric oxide synthase (nNOS)‐positive neurons is critical for normal erection because NO from its nerve endings initiates penile erection by inducing cavernous dilation.^29^ It was showed that the number of nNOS‐positive nerve fibers in the cavernosum and MPG were significantly lower in the PBS group than that in sham (Figure 6b–e). With the same Sil‐dose, the UTMD group had a relative higher nNOS level in both cavernosum and MPG than the Sil or MBs group (Figure 6b–e). It was further confirmed the accordant high expression of nNOS in the corpus cavernosum and MPG of the UTMD group by using Western blot, which almost reached the level to the sham (Figures 6f–h, S9, and S10). To further verify the neuroprotective effect in different groups, qRT‐PCR tests were performed to evaluate the expression levels of various NFs related to parasympathetic. The expression of NFs (brain‐derived neurotrophic factor [BDNF], glial cell‐derived nutrient factor [GDNF], and leukemia inhibitory factor [LIF]) were significantly improved in the UTMD group than those of PBS, Sil, and MBs groups (Figure 6i). Interestingly, NT‐3 was most significantly elevated to the Sil group of this study (Figure 6i), which may be related to the fact that Sil also has neurodegenerative properties,^30^ and the lower expression of NT‐3 in the UTMD group may be related to the fact that the number of biological replicates in this study was only six. These results indirectly demonstrated that functional penis in the UTMD group was effectively restored at least partly because of its more abundant expression of NFs.

### 3.8 Sil‐NO‐MBs combined with UTMD reduced apoptosis of CCSMCs and reversed CCF

**FIGURE 7:**
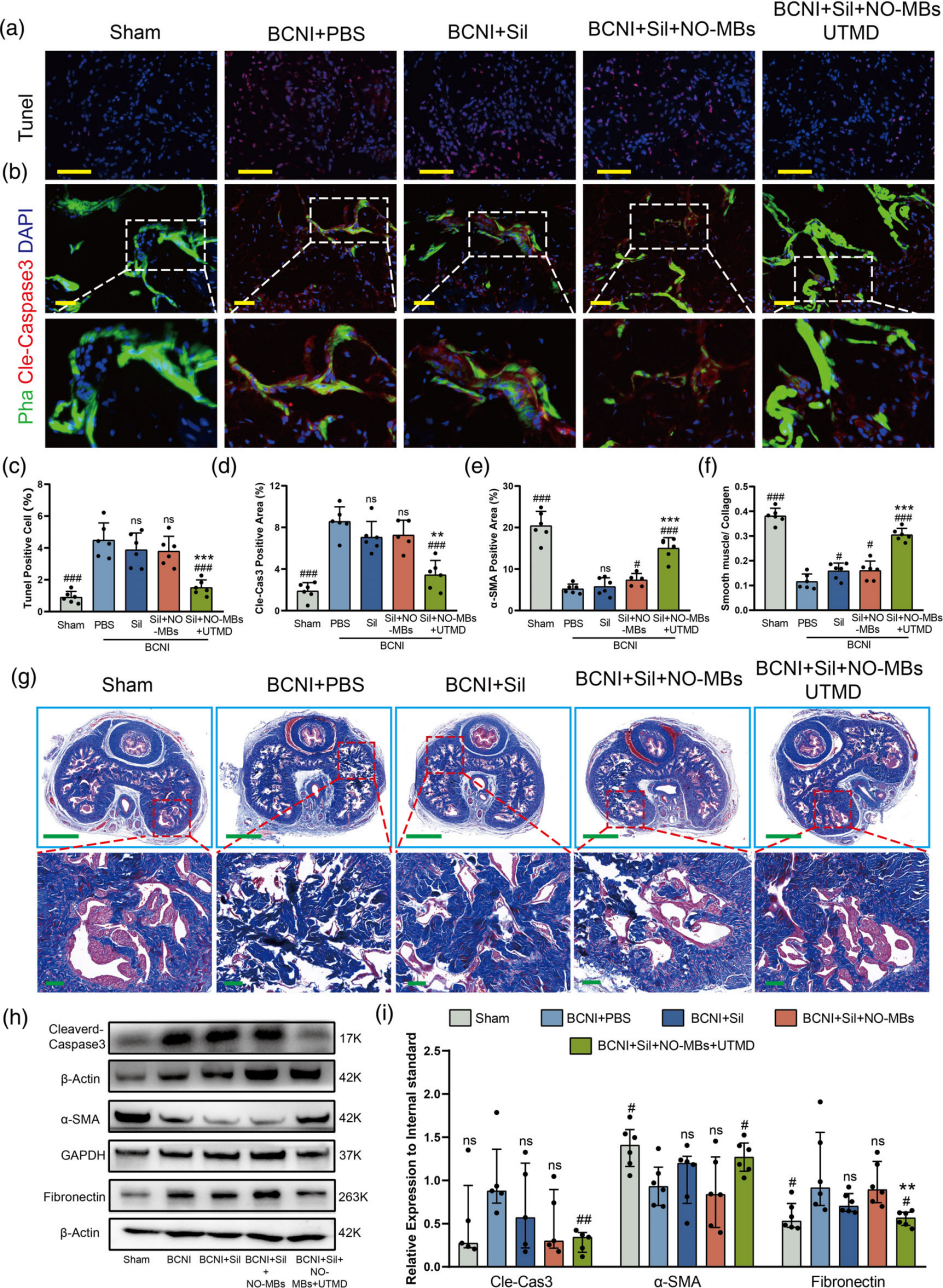
Sil‐NO‐MBs combined with UTMD reduced apoptosis of CCSMCs and reversed CCF. (a) Representative of apoptotic cells in the cavernosum were detected by means of TUNEL assay at 2 weeks after intervention, scale bars: 50 μm. (b) α‐SMA (green), Cle‐Cas3 (red), and DAPI (blue) immunostaining in cavernosum after 2 weeks of treatments, scale bars: 50 μm. (c) Quantification of apoptotic cells in the cavernosum (*n* = 6 per group). Quantification of (d) Cle‐Cas3‐ and (e) α‐SMA‐positive area by ImageJ (*n* = 6 per group). (f) Quantification of Masson's trichrome staining by the ratio of SMA‐positive area (res color) to Collagen positive area (blue color; *n* = 6 per group). (g) Representative cavernosum histology of Masson's trichrome staining, scale bars: top, 1000 μm; bottom, 100 μm. (h) Representative Western blots for Cle‐Cas3, α‐SMA, and fibronectin. (i) Normalized band intensity values (*n* = 5–6 per group). PBS versus other groups: #*p <* 0.05, ##*p <* 0.01, ###*p <* 0.001. MBs versus Sil and UTMD group: *p < 0.05, **p < 0.01, ***p < 0.001. CCF, corpus cavernosum fibrosis; CCSMCs, corpus cavernosum smooth muscle cells; Cle‐Cas3, Cleaverd‐Caspase3; DAPI, 4',6‐diamidino‐2‐phenylindole; Sil, sildenafil; α‐SMA, α‐smooth muscle antibody; UTMD, ultrasound‐targeted microbubble destruction.

CCF is an important pathological change due to neurogenic hypoxia and is characterized by diffuse fibrosis in the corpus cavernosum.^31^, ^32^ A high rate of CCSMCs apoptosis has been recognized as a critical starting step of CCF.^33^ As evidenced by TUNEL assay, the BCNI‐induced rat in the UTMD group exhibited the lowest levels of apoptosis in cavernosum (Figure 7a,c), which was in accordance with alleviation of apoptosis in CCSMCs (Figure 7b–e). Accordingly, the least CCF in the UTMD were shown which nearly recovered to the sham in comparison to PBS, Sil and MBs group (Figure 7f,g). Interestingly, a mild reduction of CCSMCs apoptosis and slight improvement of CCF were also observed in neurogenic rats receiving Sil and MBs, indicating that the spontaneous rupture of MBs that occurs in penile tissue can release small amounts of NO, there is evidence that NO actually acts as an antiapoptotic agent, depending on the tissue and physiological conditions.^34^ In addition, there was also an obvious decrease of both fibronectin (a protein associated with fibrous deposition) and cleaved caspase‐3, but significantly increase of α‐SMA (marker of cavernous smooth muscle) in the UTMD group in comparison to that of the PBS, Sil, and MBs group (Figures 7h,i and S11–S13). These results implied that Sil‐NO‐MBs + UTMD effectively reduced CCSMCs apoptosis and reversed CCF in neurogenic rats.

## 4 DISCUSSION

The purpose of this study was to explore whether MBs loaded with PDE5i and NO accompanied with UTMD could effectively antagonize adverse remodeling of corpus cavernosum for regained erection in neurogenic ED. The results clearly showed that MBs combined with UTMD could protect the penis from hypoxia and oxidative stress in cavernosum induced by poor microvascular perfusion. In addition, more NFs were produced in cavernosum under the joint intervention, and recovered the integrity of the CN. Besides, multifunctional MBs cooperated with UTMD could inhibit the apoptosis of CECs and CCSMCs, and reversed CCF after denervation. It indicated that the poor remodeling of penis after denervation had been effectively reversed under coordinated intervention, importantly, the erectile function in the model rats was recovered at different degrees (details are shown in Scheme 1). Together, this study provides the first evidence that Sil‐NO‐MBs combined with UTMD is beneficial to the treatment of neurogenic ED.

George Han reports that nanoparticles encapsulating the erectogenic agents, including tadalafil, sialorphin, and NO were applied as a gel to the glans, which could increase erectile function in a rat model of aging ED.^35^ Moses Tar found that topically applied NO‐releasing nanoparticles can elicit erections in a neurogenic rat model.^36^ However, as an extremely unstable gas, NO is easy to be oxidized into toxic gas nitrogen dioxide in the external environment, and it may be insufficient to release enough PDE5i on the skin surface to the cavernous interstitium to exert its efficacy. Here, we successfully prepared MBs loaded with Sil and NO, and achieved targeted therapeutic molecular release through UTMD while ensuring the stability of NO in MBs. First, PDE5i was encapsulate phospholipid layer of MBs and NO was embedded in the gas core of MBs simultaneously with favorable stability. Second, after MBs injection followed by UTMD application, the cavitation effect appeared which on one hand induced the collapsed of the MBs and on the other hand promoted the permeability of cell membrane. Ultimately, abundant PDE5i and NO released from MBs possessed more possibilities entering into targeted cells. Consequently, this approach not only benefit for enhancing local PDE5i concentration at a lower dose, but also ensures considerable NO concentration which further contributing for penis protection.

There is no timely and accurate method to evaluate the occurrence and development of neurogenic ED, and erectile reaction alone cannot fully reflect the efficacy of drugs, because the recovery of erectile reaction generally occurs in the later stage of tissue protection. In this research, such therapeutic MBs also owned ultrasound imaging ability which is similar to clinical used MBs (e.g., Sonovue) in size and structure.^37^ Notably, ULM has emerged as a novel microvascular imaging tool that can noninvasively visualizing capillary‐scale microvessels in vivo at clinically relevant penetration depths recently.^38^, ^39^ Therefore, we performed the ULM imaging basing on Sil‐NO‐MBs at the endpoint of the therapy course to observe the microcirculation of corpus cavernosum. As a result, the microvascular density depicted by ULM showed consistent tendency with pathological PECAM‐1 which has been used as a specific marker of endothelial cell. We believe that our application strongly recommends a new indication for ULM which offers novel insight into noninvasive evaluation of neurogenic ED in the future.

The drug encapsulation rate obtained in this study was maintained at about 50% using 2 mg of Sildenafil for drug delivery. Similar to the dose used in Doreen E's study and other studies,^40^ the total amount of Sil in all groups of this study was intervened at 1 mg/kg which was considered effective for penile response and avoiding adverse structural remodeling of the target organ instead of the transient pursuit of sexual intercourse only. Notably, the effective dose of Sil in our study is also much lower than the daily sild dose corrected for differences in human surface area (20 mg/kg body weight),^41^ which is particularly advantageous in avoiding unpredictable and serious side effects brought by repeated utilization of high doses. However, it is undeniable that this study is a conceptual illustration of an innovative application of PDE5i and does not yet represent the optimal dose for subsequent clinical translation. In addition, to avoid direct pharmacological reactions from the drug, we performed erectile function assessment and histological testing 48 h after the last dose, a time span in which there was already hardly any in vivo drug retention, since the half‐life of Sil in humans is at least three times longer than in rats (0.4–1.3 h in rats compared to 4 h in humans).^42^ It should be noted that the illustration with Sil‐NO‐MBs combined with UTMD did not affect activity of CCSMCs and did not cause pathological damage to the penis and other vital organs (Figures S3 and S4), which supports the safety of the method.

It cannot be ignored that there are still some limitations in our study. The primary observation in this study was early penile rehabilitation, while longer‐lasting penile protection and recovery of erectile function after treatment were not observed. Further studies are needed to test whether repeated intravenous injections of Sil‐NO‐MBs combined with UTMD at the penis would induce a more durable recovery of erectile function. Notably, the efficacy of Sil‐NO‐MBs + UTMD in human neurogenic ED remains to be further evaluated, as rodent models have high regenerative potential compared to large animals and humans. We are preparing to apply the intervention model of Sil‐NO‐MBs + UTMD in a nonhuman primate model of neurogenic ED, which will contribute to the clinical translation of this technology.

## 5 CONCLUSION

In summary, we prepared MBs loaded with both Sil and NO in combination with UTMD to achieve accurate release of Sil and NO in cavernous tissue in this study. This approach effectively reversed the poor structural remodeling of the penis by increasing microvascular perfusion, avoiding apoptosis of cavernosum, protecting nNOS‐positive neurons and reversing CCF in neurogenic ED, and ultimately regained penile erection. Our work subtly proposes a concept of “awakening” the efficacy of PDE5i, and we believe this manner will encourage more researchers to further explore innovative treatment options for neurogenic ED.

## AUTHOR CONTRIBUTIONS

**Wende Yang:** Project administration (lead); software (lead); writing – original draft (lead). **Chen Qiu:** Data curation (lead); project administration (equal); software (equal); writing – review and editing (lead). **Jiancheng Zhai:** Methodology (equal); project administration (equal); software (equal). **Wei Zhang:** Data curation (equal); methodology (equal); software (equal). **Chengwu Huang:** Methodology (equal); software (equal). **Jun Shao:** Methodology (supporting); project administration (supporting); software (supporting). **Jingke Zhang:** Data curation (supporting); methodology (equal); software (supporting). **Shigao Chen:** Project administration (supporting); software (supporting); visualization (supporting). **Xiaoyan Miao:** Methodology (supporting); project administration (supporting); software (supporting). **Peng Chen:** Methodology (supporting); project administration (supporting); software (supporting). **Bo Wei:** Conceptualization (lead); funding acquisition (equal); supervision (lead); validation (equal). **Jie Ren:** Conceptualization (lead); funding acquisition (equal); resources (equal); supervision (lead). **Hongbo Wei:** Conceptualization (lead); funding acquisition (lead); resources (lead); validation (lead); writing – review and editing (equal).

## CONFLICT OF INTEREST STATEMENT

The authors declare no conflicts of interest. The authors declare that the figure of Schematic was designed by our team, and do not acquire from any copyrighted source, without acknowledgement.

## Supporting information


**Data S1.** Supporting Information.

## Data Availability

The data that support the findings of this study are available from the corresponding author upon reasonable request.
