# Supplementary material for: Ultrasound‐targeted microbubble destruction mediates PDE5i/NO integration for cavernosum remodeling and penile rehabilitation
Source: Bioeng Transl Med. 2023 Jul 2;8(5):e10568. doi: 10.1002/btm2.10568 (PMC10486332; doi:10.1002/btm2.10568)
Supplement: Supplementary file 1 — Data S1. Supporting Information. [file BTM2-8-e10568-s001.docx]

**Supplementary Information**

**Ultrasound-Targeted Microbubble Destruction Mediates PDE5i/NO Integration for Cavernosum Remodeling and Penile Rehabilitation**

Wende Yang^1#^, Chen Qiu^2,3#^, Jiancheng Zhai^1^, Wei Zhang^2^, Chengwu Huang^4^, Jun Shao^1^, Jingke Zhang^4^, Shigao Chen^4^, Xiaoyan Miao^2^, Peng Chen^1^, Bo Wei^1*^, Jie Ren^2*^, Hongbo Wei^1*^

^1^Department of Gastrointestinal Surgery, The Third Affiliated Hospital of Sun Yat-sen University, Tianhe Road 600, Guangzhou 510630, China.

^2^ Department of Medical Ultrasound, Laboratory of Novel Optoacoustic (Ultrasonic)

imaging, The Third Affiliated Hospital of Sun Yat-sen University, Guangzhou 510630, China.

^3^ Department of Ultrasound, Second Affiliated Hospital of Zhejiang University School of Medicine, Hangzhou, 310052, China.

^4^ Department of Radiology, Mayo Clinic College of Medicine and Science, Rochester, MN 55905, USA

^#^These authors contributed equally to this work.

* Correspondence: B.W. ([weibo3@mail.sysu.edu.cn](mailto:weibo3@mail.sysu.edu.cn)), J.R. (renj@mail.sysu.edu.cn) or to H.W. ([weihb@mail.sysu.edu.cn](mailto:weihb@mail.sysu.edu.cn)).

**Supplementary Methods**

**DLC & DLE measurement of MBs**

To determine the drug loading content (DLC) and drug loading efficacy (DLE) of PDE5i, UV absorptions of the MBs and drug were measured using the Nanodrop 2000 spectrophotometer (Nanodrop Technologies, Wilmington, USA) at 292 nm. Firstly, the standard curve of PDE5i were analyzed basing on UV absorptions of different concentration of PDE5i solutions (0, 31.25, 62.5, 125, 250 mg/ml in hydrochloric acid). Secondly, pre-weighted freeze-dried PDE5i loaded MBs were also re-dissolved in 0.5 ml of hydrochloric acid to obtain their UV absorptions. Finally, the PDE5i concentrations of MBs were calculated according to the previous standard curve. The DLC was calculated as the weight percentage of PDE5i in MBs and the DLE was calculated as the ratio of the encapsulated PDE5i weight to the total PDE5i weight.

**Measurement of coumarin and NO in CCSMCs**

Intracellular NO concentration was detected using 3-Amino,4-aminomethyl-2',7'-difluorescein, diacetate (DAF-FM DA) (Beyotime, S0019) fluorescent probe method, divided into experimental group: Sil-NO-MBs + UTMD; control group: Sil-NO-MBs were given only. Images were collected under fluorescence microscope after washing with PBS 3 times at the end of the intervention. Coumarin-NO-MBs were prepared by replacing Sildenafil with coumarin. CCSMCs were evenly grown in six-well plates, experimental group: coumarin-NO-MBs + UTMD, control group: no other treatment after adding coumarin-NO-MBs. Images were collected under fluorescence microscope after washing 3 times with PBS. Quantitative analysis of the collected images was performed using ImageJ software.

**Serum analysis**

Serum liver function indicators including alanine aminotransferase (ALT), aspartate aminotransferase (AST), renal function indicators: urea nitrogen (BUN), creatinine (Scr), cardiac function indicators: creatine kinase (CK) and creatine kinase isoenzyme (CK-MB) were measured using a fully automated biochemical analyzer (Chemray 240/800, Rayto).

**Supplementary Figures and Tables**


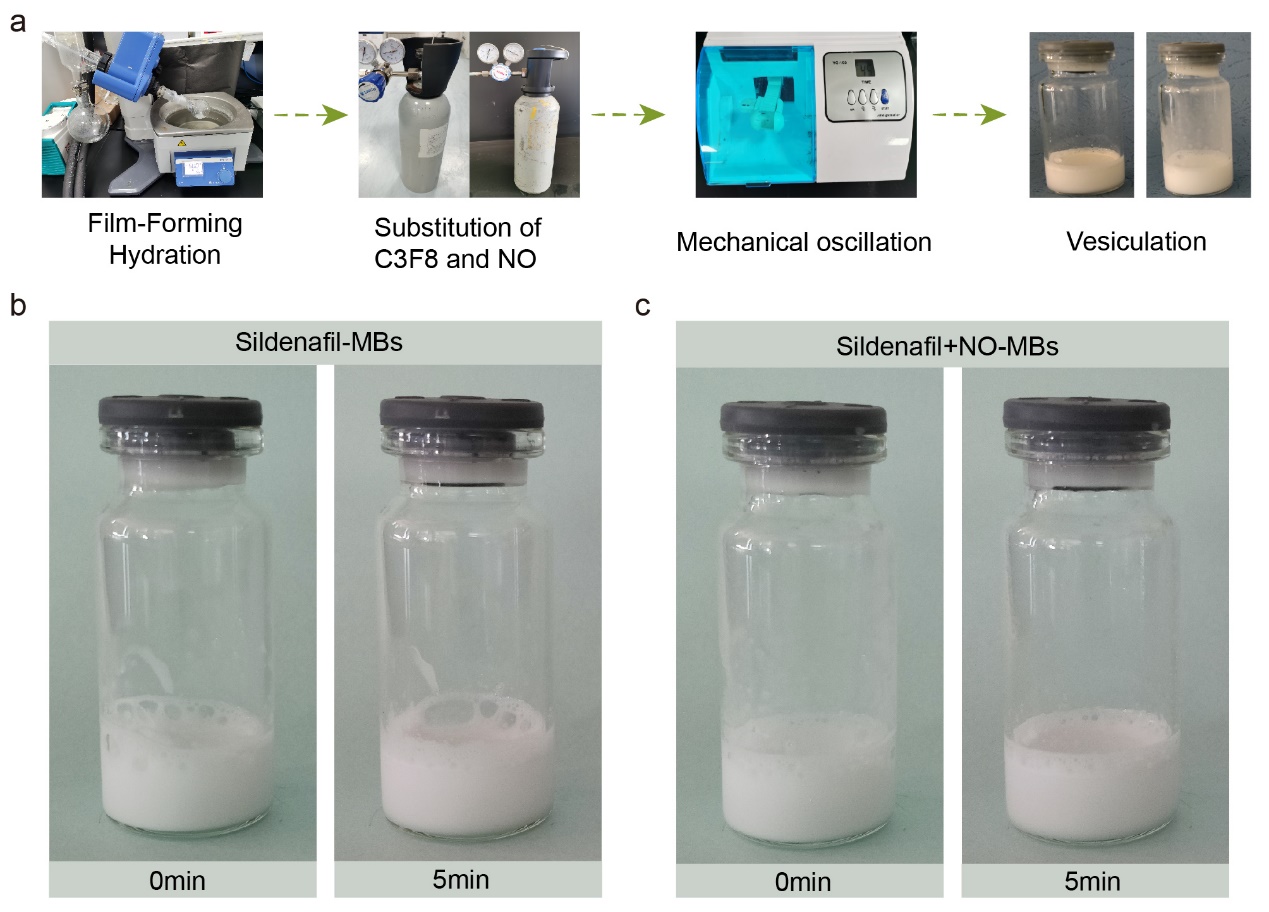


**Supplementary Figure 1. (a)** A brief schematic diagram of microbubble fabrication. **(b, c)** Broad diagram of microbubbles prepared with or without NO at 2 mg sildenafil.


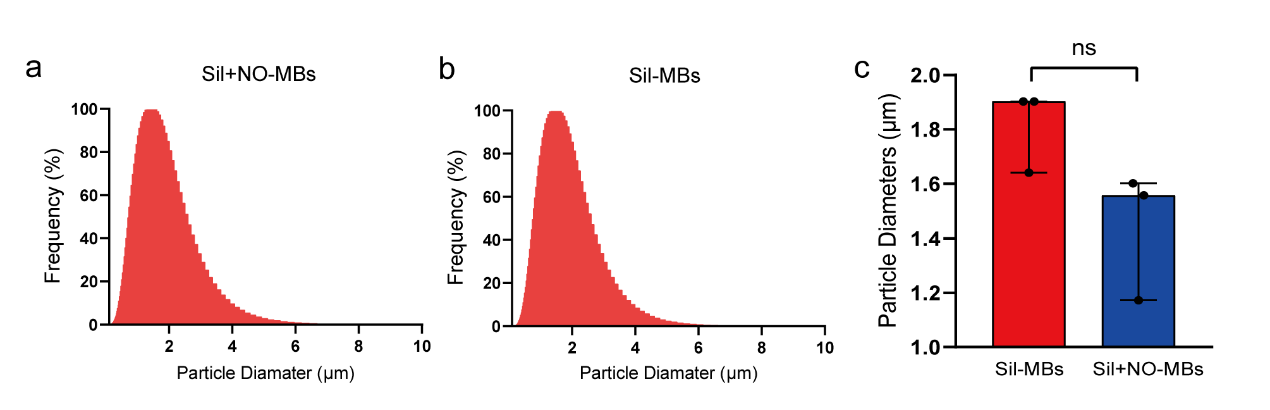


**Supplementary Figure 2. (a, b)** Particle size analysis with or without NO microvesicles under 2 mg sildenafil was performed and **(c)** quantified.


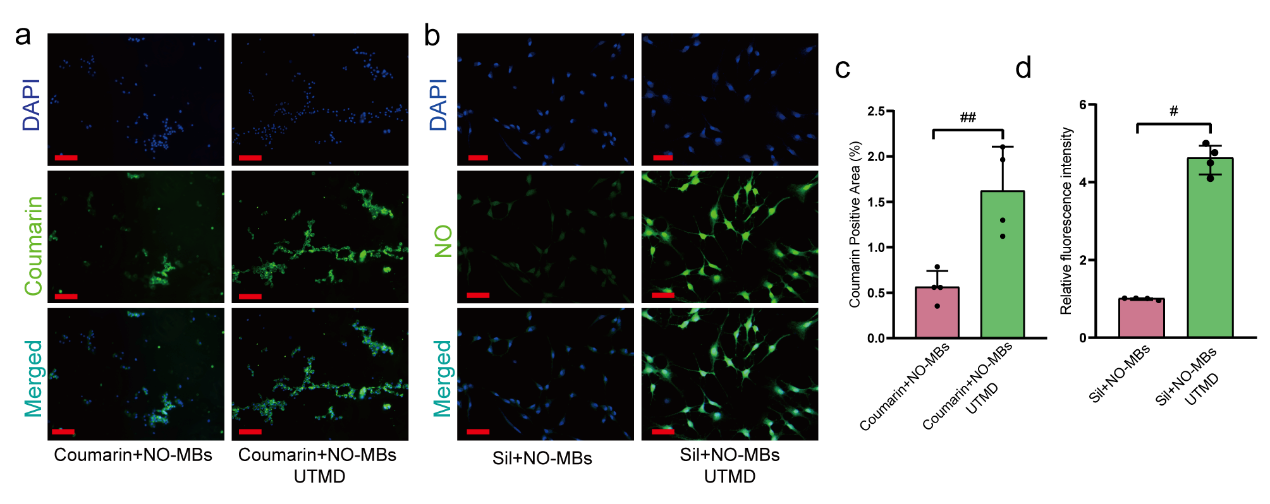


**Supplementary Figure 3.** Promotion of internalization of drug and gas by CCSMCs in vitro under UTMD. **(a)** Green fluorescence (Coumarin) expression of CCMSCs in the presence or absence of UTMD with Coumarin-NO-MBs, Scale bar=100 μm. **(b)** Determination of NO (green fluorescence) expression in CCSMCs in the presence or absence of UTMD in Sil-NO-MBs by DAF-FM DA method, Scale bar=50 μm. Quantification of fluorescence intensity of Coumarin **(c)** and NO **(d)** in CCSMCs (n=3 per group). ^##^*p*＜0.01, ^###^*p*＜0.001.


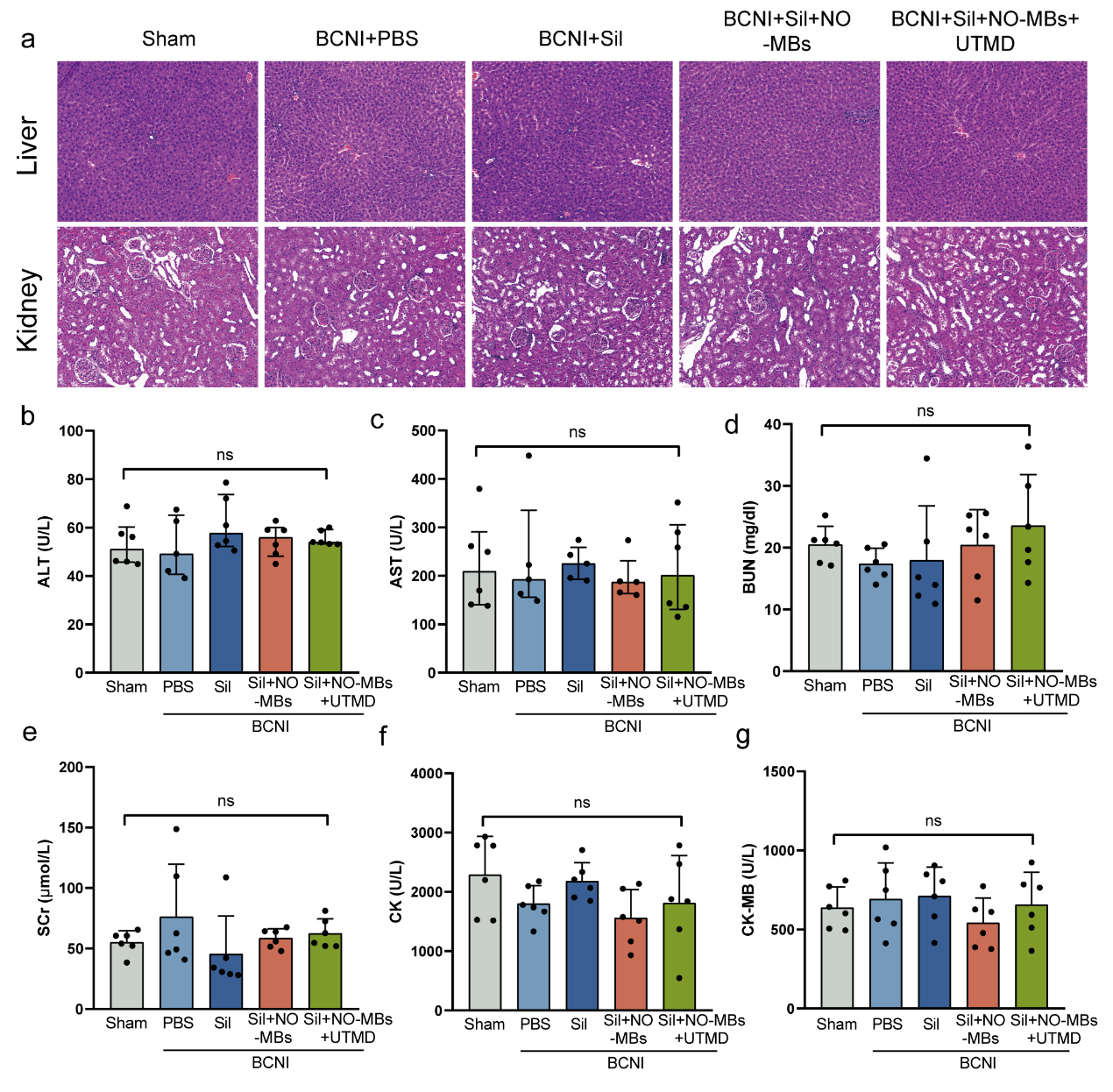


**Supplementary Figure 4.** In vivo organ side effect assessment. **(a)** HE staining was to evaluate the liver and kidney structure of rats in each group after intervention. **(b-g)** Collect the serum of rats in each group, and use the biochemical analyzer to evaluate the heart, liver and kidney functions of rats in each group after the intervention (n=6 per group). Abbreviations: ALT, alanine aminotransferase; AST, aspartate aminotransferase; BUN, urea nitrogen; Scr, creatinine; CK, creatine kinase; CK-MB, creatine kinase isoenzyme.


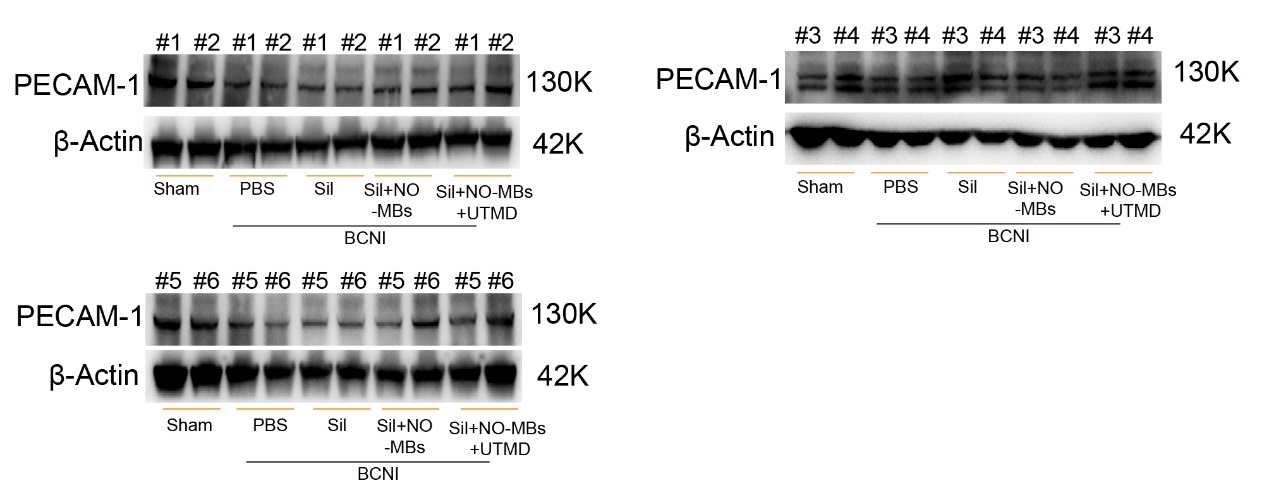


**Supplementary Figure 5.** Duplicate data of PECAM-1 through western blot.


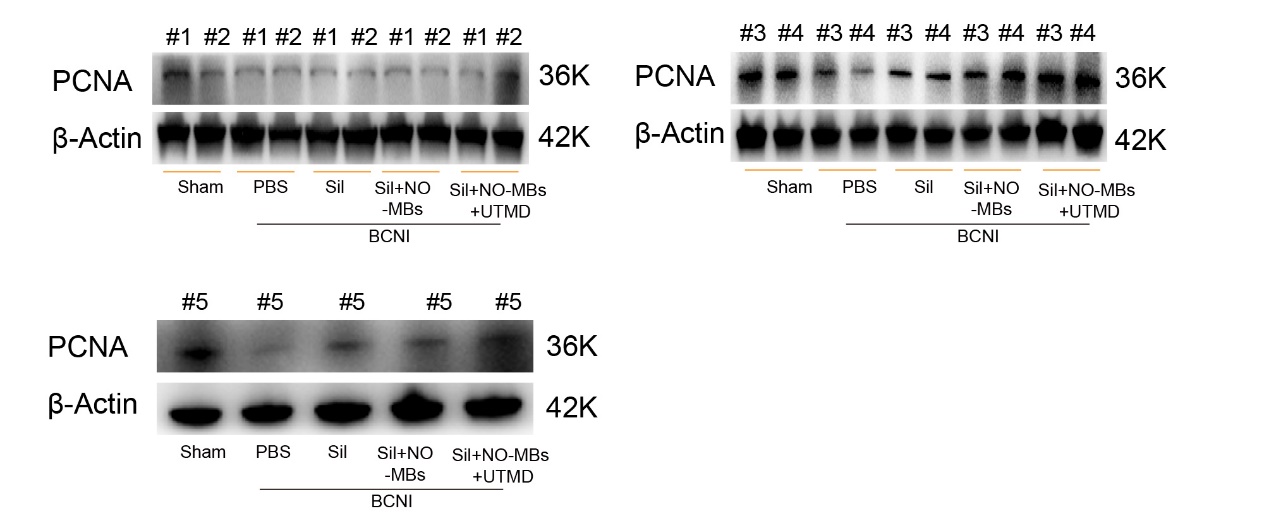


**Supplementary Figure 6.** Duplicate data of PCNA through western blot.


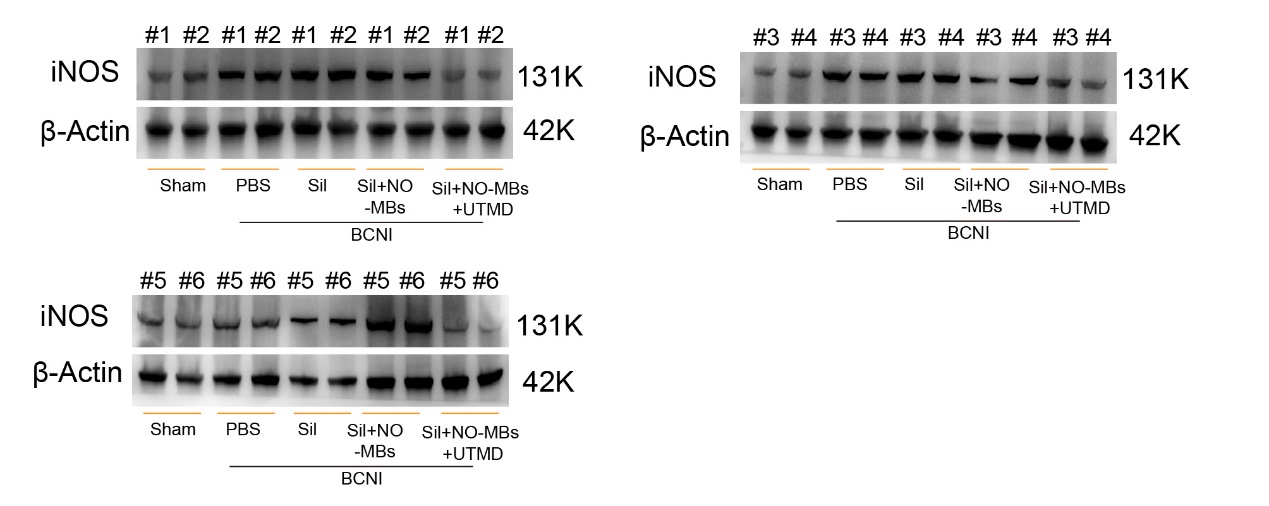


**Supplementary Figure 7.** Duplicate data of iNOS through western blot.


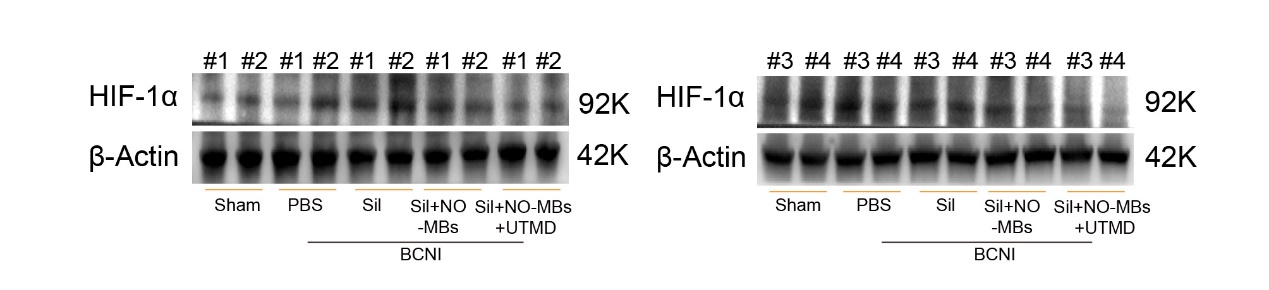


**Supplementary Figure 8.** Duplicate data of HIF-1α through western blot.


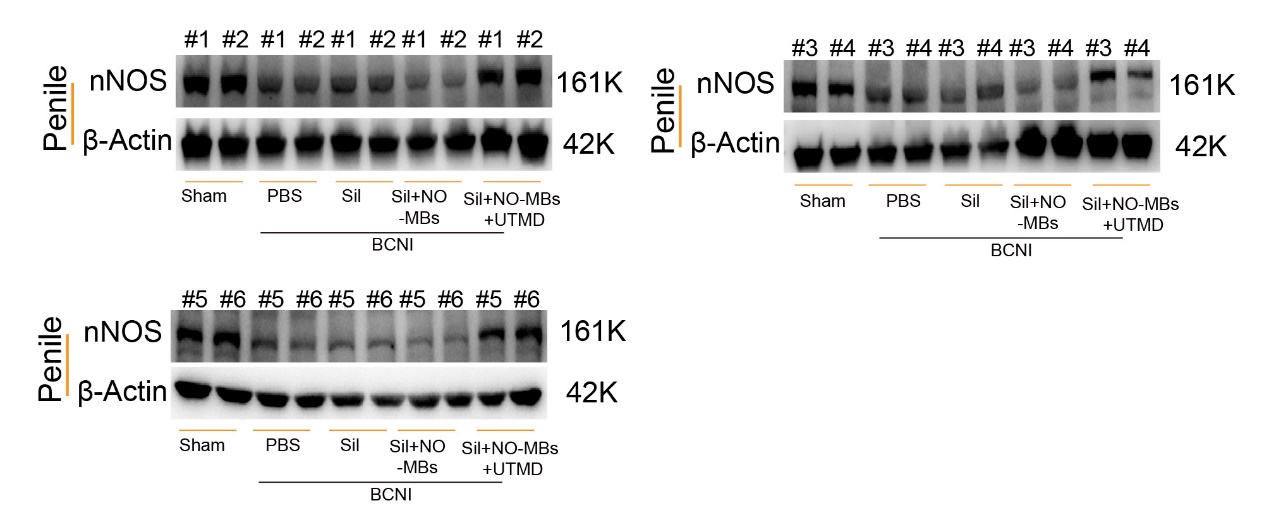


**Supplementary Figure 9.** Duplicate data of nNOS in penis through western blot.


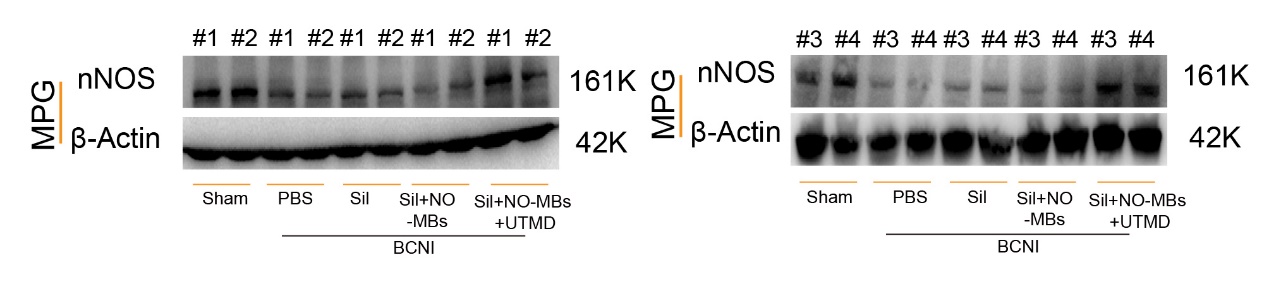


**Supplementary Figure 10.** Duplicate data of nNOS in MPG through western blot.


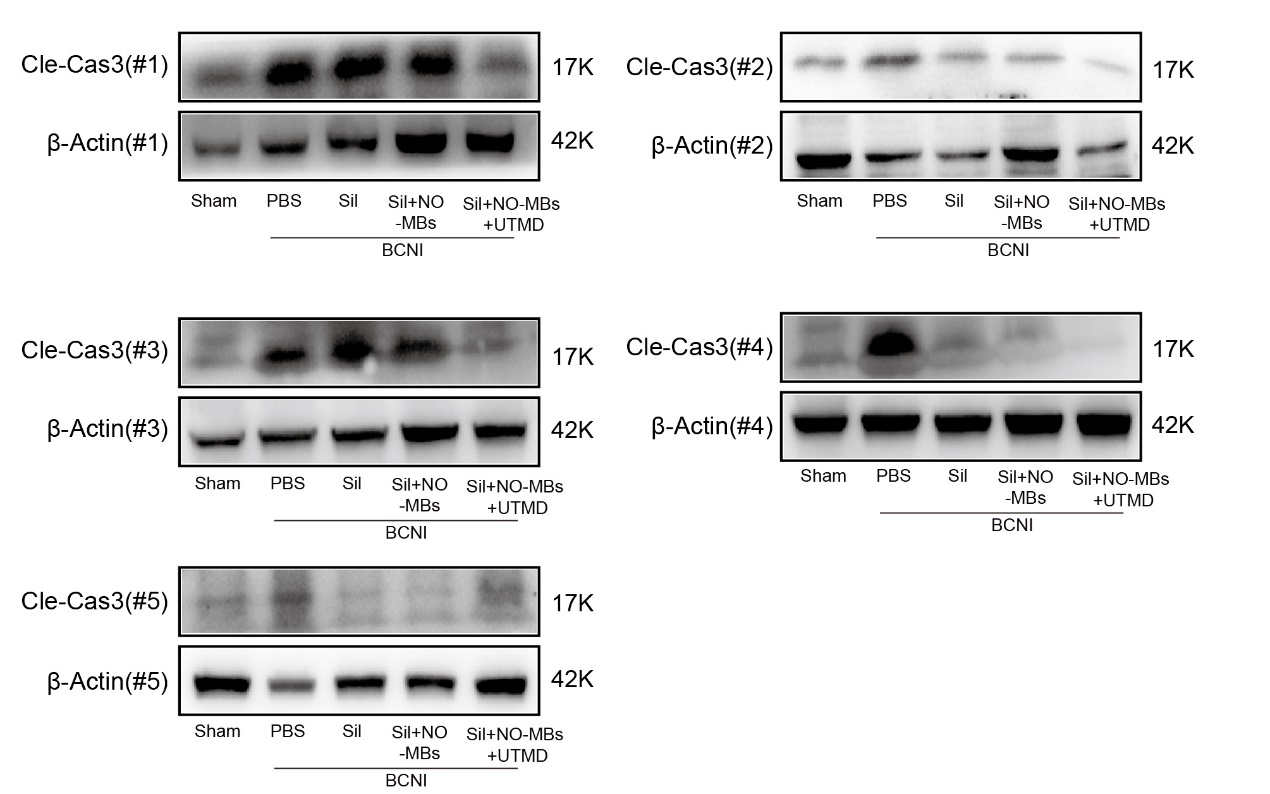


**Supplementary Figure 11.** Duplicate data of Cle-cas3 through western blot.


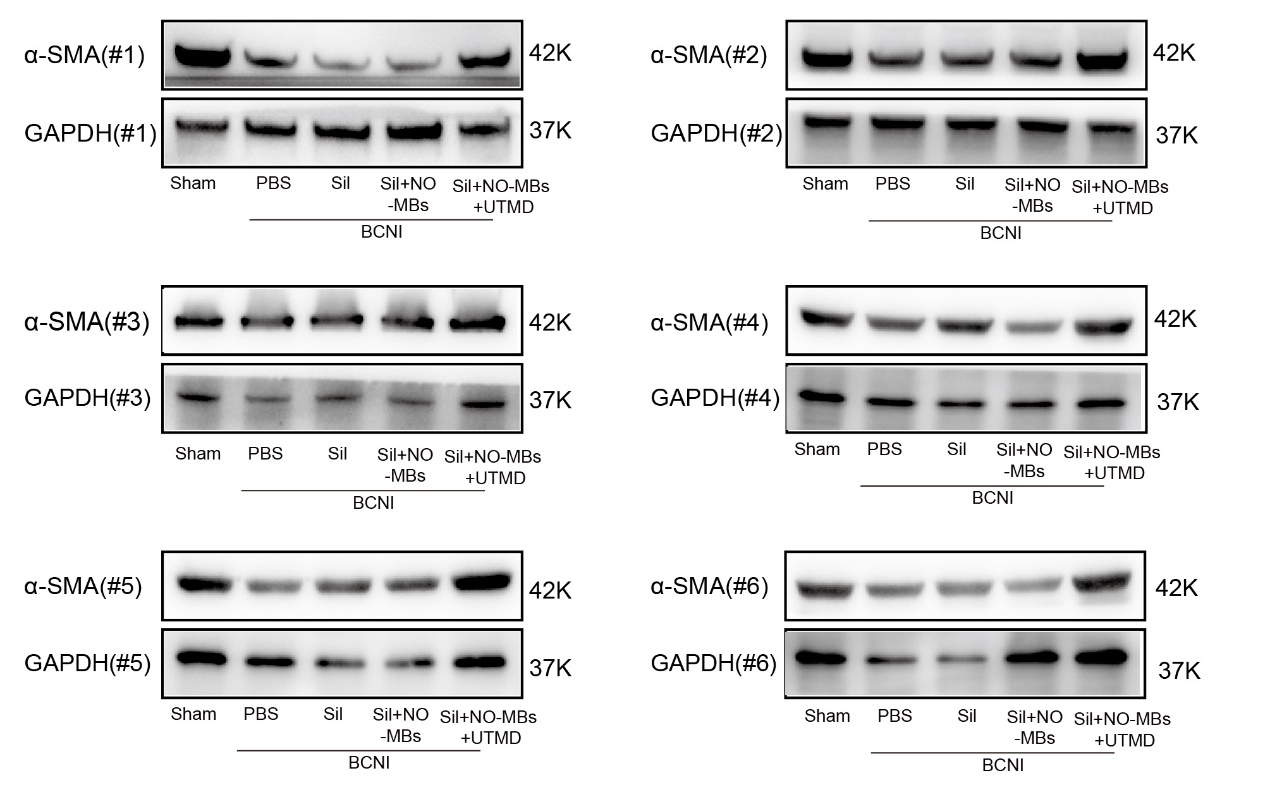


**Supplementary Figure 12.** Duplicate data of α-SMA through western blot.


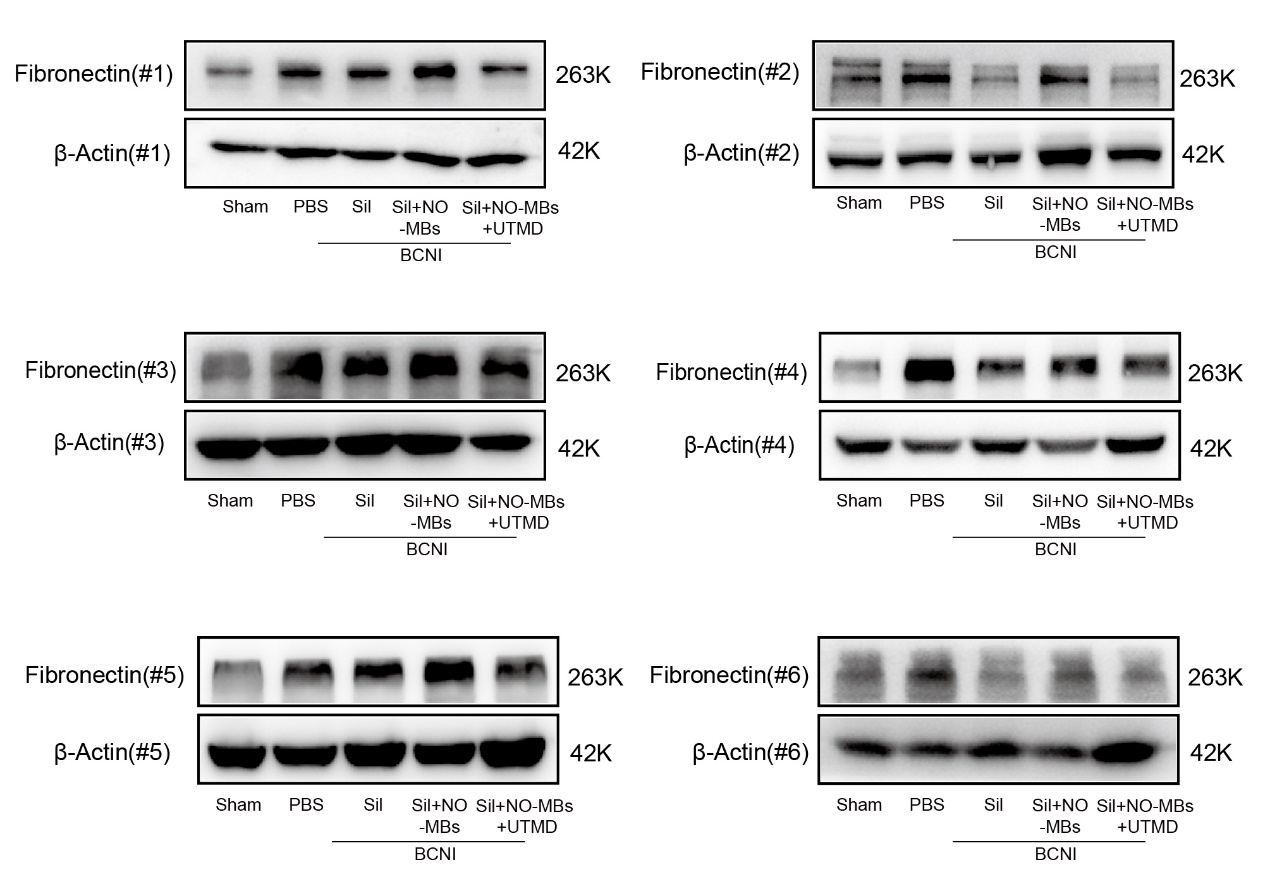


**Supplementary Figure 13.** Duplicate data of Fibronectin through western blot.

| Table S1 - Primer sequences of each gene. | | |
| --- | --- | --- |
| Target | Forward | Reverse |
| BDNF | TGGAACTCGCAATGCCGAACTAC | TCCTTATGAACCGCCAGCCAATTC |
| GDNF | CGCTGACCAGTGACTCCAATATGC | AGTGCCGCCGCTTGTTTATCTG |
| LIF | CCTTCCCATCACCCCTGTAAATGC | TGCCGTTGAGTTGAGCCAGTTG |
| NT-3 | CGGAGTTCGGCATGGCTATTGG | ACACTCTTCCTCCTCGGCTTCC |
| GAPDH | CCTGGAGAAACCTGCCAAGTAT | TAGCCCAGGATGCCCTTTAGT |

| Table S2 - Ratio of ICP/MAP and peak ICP in the different groups. | | |  |
| --- | --- | --- | --- |
| Group | ICP/MAP  (Mean ± SD) | Peak ICP (mmHg)  (Mean ± SD) |  |
| Sham | 0.81±0.13 | 101.12±8.38 |  |
| BCNI+PBS | 0.25±0.01 | 37.67±3.10 |  |
| BCNI+Sil | 0.33±0.02 | 62.95±1.91 |  |
| BCNI+Sil-NO-MBs | 0.39±0.02 | 61.04±1.41 |  |
| BCNI+Sil-NO-MBs+UTMD | 0.65±0.06 | 86.29±3.30 |  |

| Table S3 - Related information about primary antibodies | | | | | |  |
| --- | --- | --- | --- | --- | --- | --- |
| Catalog  Number | Product  Name | Species  Reactivity | Dilution  (WB/IF or IHC) | Molecular  weight | Source |  |
| 19677-1-AP | Cle-Caspase3 | Rabbit | 1:1000/1:250 | 17 kDa | Proteintech, America |  |
| A2104 | PECAM-1 | Rabbit | 1:1000 | 130 kDa | ABclonal, China |  |
| AF0239 | PCNA | Rabbit | 1:1000/1:200 | 36 kDa | Affinity Biosciences, America |  |
| 18985-1-AP | iNOS | Rabbit | 1:1000/1:200 | 131 kDa | Proteintech, America |  |
| AB179483 | HIF-1α | Rabbit | 1:1000 | 92 kDa | Abcam, England |  |
| AF1009 | HIF-1α | Rabbit | -/1:100 | - | Affinity Biosciences, America |  |
| GTX634455 | nNOS | Mouse | 1:1000 | 161 kDa | GeneTex, America |  |
| 4231S | nNOS | Rabbit | -/1:200 | - | Cell Signaling Technology, America |  |
| BF9212 | α-SMA | Mouse | 1:1000 | 42 kDa | Affinity Biosciences, America |  |
| AB45688 | Fibronectin | Rabbit | 1:3000 | 263 kDa | Abcam, England |  |
| G1028 | Pha | - | -/1:250 | - | Servicebio, China |  |
| AC001 | GAPDH | Rabbit | 1:5000 | 37 kDa | ABclonal, China |  |
| 8457S | β-Actin | Rabbit | 1:1000 | 42kDa | Cell Signaling Technology, America |  |
